# Distinct Screening Approaches Uncover PA14_36820 and RecA as Negative Regulators of Biofilm Phenotypes in Pseudomonas aeruginosa PA14

**DOI:** 10.1128/spectrum.03774-22

**Published:** 2023-03-27

**Authors:** Amal H. Yahya, Sophie R. Harston, William L. Colton, Matthew T. Cabeen

**Affiliations:** a Department of Microbiology and Molecular Genetics, Oklahoma State University, Stillwater, Oklahoma, USA; Emory University School of Medicine

**Keywords:** *Pseudomonas aeruginosa*, RecA, biofilms, recombination

## Abstract

Pseudomonas aeruginosa commonly infects hospitalized patients and the lungs of individuals with cystic fibrosis. This species is known for forming biofilms, which are communities of bacterial cells held together and encapsulated by a self-produced extracellular matrix. The matrix provides extra protection to the constituent cells, making P. aeruginosa infections challenging to treat. We previously identified a gene, *PA14_16550*, which encodes a DNA-binding TetR-type repressor and whose deletion reduced biofilm formation. Here, we assessed the transcriptional impact of the *16550* deletion and found six differentially regulated genes. Among them, our results implicated *PA14_36820* as a negative regulator of biofilm matrix production, while the remaining 5 had modest effects on swarming motility. We also screened a transposon library in a biofilm-impaired Δ*amrZ* Δ*16550* strain for restoration of matrix production. Surprisingly, we found that disruption or deletion of *recA* increased biofilm matrix production, both in biofilm-impaired and wild-type strains. Because RecA functions both in recombination and in the DNA damage response, we asked which function of RecA is important with respect to biofilm formation by using point mutations in *recA* and *lexA* to specifically disable each function. Our results implied that loss of either function of RecA impacts biofilm formation, suggesting that enhanced biofilm formation may be one physiological response of P. aeruginosa cells to loss of either RecA function.

**IMPORTANCE** Pseudomonas aeruginosa is a notorious human pathogen well known for forming biofilms, communities of bacteria that protect themselves within a self-secreted matrix. Here, we sought to find genetic determinants that impacted biofilm matrix production in P. aeruginosa strains. We identified a largely uncharacterized protein (PA14_36820) and, surprisingly, RecA, a widely conserved bacterial DNA recombination and repair protein, as negatively regulating biofilm matrix production. Because RecA has two main functions, we used specific mutations to isolate each function and found that both functions influenced matrix production. Identifying negative regulators of biofilm production may suggest future strategies to reduce the formation of treatment-resistant biofilms.

## INTRODUCTION

Pseudomonas aeruginosa is a Gram-negative opportunistic pathogen of significance to human health, especially for immunocompromised patients and individuals with cystic fibrosis (1). P. aeruginosa can be difficult to treat due to its many virulence factors and behaviors, including biofilm formation (2). Biofilms comprise a community of bacterial cells that are held together and encapsulated by a self-produced extracellular matrix. The biofilm matrix provides extra protection to P. aeruginosa cells, allowing them to tolerate harsh environmental conditions and resist antibiotics (3). Production of the biofilm matrix also is associated with biofilm phenotypes such as adhesion to biotic and abiotic surfaces in industrial (4) and medical settings (5). One readily visible phenotype associated with biofilm formation in many bacterial species is colony wrinkling. Colony wrinkling as a proxy for biofilm formation has long been reported in many bacteria, including P. aeruginosa (6), Bacillus subtilis (7), and Escherichia coli (8). On certain solid media, biofilm-forming strains display a wrinkled colony morphology, whereas non-biofilm formers, or strains defective for biofilms, show a smooth morphology. We have previously used this characteristic of biofilm-forming strains to identify genes that influence biofilm production (9).

Biofilm formation is a sequential process that is initiated by planktonic (free-swimming) bacteria landing on and then attaching to a surface; conversion from reversible to irreversible attachment is commonly accompanied by developmental changes, including alterations to flagellar function to decrease swimming (10), production of type IV pili to aid with attachment (11), intracellular accumulation of the second messenger cyclic dimeric GMP (c-di-GMP) (12), and increased production of exopolysaccharides (EPS) and adhesins (13). P. aeruginosa has multiple modes of motility, which include swimming and twitching motility (14). Numerous previous reports have highlighted the importance of swimming motility for biofilm formation in P. aeruginosa and in other organisms (15–19). Swimming by P. aeruginosa is mediated by its polar single flagellum, whereas twitching is mediated by type IV pili (20, 21).

EPS are a major component of the biofilm matrix in P. aeruginosa and have been reported to have a role in attachment and virulence (22–24). The principal biofilm EPS of P. aeruginosa strain PA14 is Pel, whose production depends on a seven-gene operon (25). EPS are particularly important for biofilm maturation and the structural stability of mature biofilms. Colvin et al. (22, 26) demonstrated that disturbing EPS synthesis significantly arrested biofilm maturation, as Pel-deficient strains did not grow past the monolayer stage of biofilm development and appeared as smooth colonies, consistent with poor biofilm formation. Additionally, Jennings et al. showed that Pel is a cationic exopolysaccharide that is important for cross-linking extracellular DNA, another important component of the biofilm matrix, thereby contributing to the overall mechanical strength of P. aeruginosa biofilms (27). Pel synthesis is regulated both transcriptionally and posttranslationally. Its synthesis is mainly regulated by c-di-GMP (28–30). c-di-GMP is important for coordinating the lifestyle transition from planktonic to biofilm and is synthesized by diguanylate cyclases (DGCs) and degraded by phosphodiesterases (PDEs) (31). At elevated levels of c-di-GMP, c-di-GMP directly binds to a c-di-GMP-responsive repressor called FleQ, causing derepression of the *pel* operon (29). Posttranslationally, PelD, one of the enzymes responsible for Pel synthesis, is directly regulated by c-di-GMP binding (30, 32). The current model suggests that high intracellular levels of c-di-GMP are associated with biofilm formation, whereas low c-di-GMP levels are associated with a planktonic lifestyle (33).

We recently identified the PA14_16550 protein, a TetR-type DNA repressor, as influencing biofilm formation in P. aeruginosa. Deletion of *16550* significantly decreased the production of EPS and lowered cyclic di-GMP levels (9) in multiple biofilm-overproducing strain backgrounds, including a moderately hyper-biofilm-forming strain called Δ*amrZ* (9, 34). AmrZ is a global transcriptional regulator that regulates many key phenotypes, including by repressing cyclic di-GMP and exopolysaccharide production (35). The mechanism by which 16550 regulates biofilm formation and/or cyclic di-GMP levels has remained unknown. However, the P. aeruginosa PAO1 ortholog of 16550, PA3699, is 100% identical to 16550 and was first identified as binding to the promoter of the *lasR* gene, experimentally establishing 16550 as a DNA-binding protein (36). PA3699 was characterized as a repressor of *lasR*, which encodes a quorum-sensing regulator, but only when PA3699 was overproduced; there was no detectable quorum-related phenotype when the *PA3699* gene was deleted from the chromosome (36). From these data, we inferred that the biofilm suppression we observed upon deletion of *16550* (9) is a separate phenomenon from any interaction with *lasR*. Given that 16550 is a DNA-binding protein, we hypothesized that deletion of *16550* may suppress biofilm formation in PA14 because of altered expression of biofilm-related genes.

Here, we used transcriptomic analysis to examine changes in global gene expression due to deletion of *16550*, and we found significant changes in the expression of a relatively small group of genes. After analyzing the biofilm effects resulting from deletion or overexpression of these genes, we took a mutagenesis and visual screening approach to find genes whose deletion could reverse biofilm suppression in a Δ*16550* strain. Surprisingly, we identified the recombination protein-encoding gene *recA*. Because RecA functions both in homologous recombination and in the SOS response, we genetically dissected these functions and assessed their individual contributions to biofilm phenotypes; we found that abrogation of either function increased biofilm levels.

## RESULTS

### Impact of *16550* deletion on gene expression.

**FIG 1:**
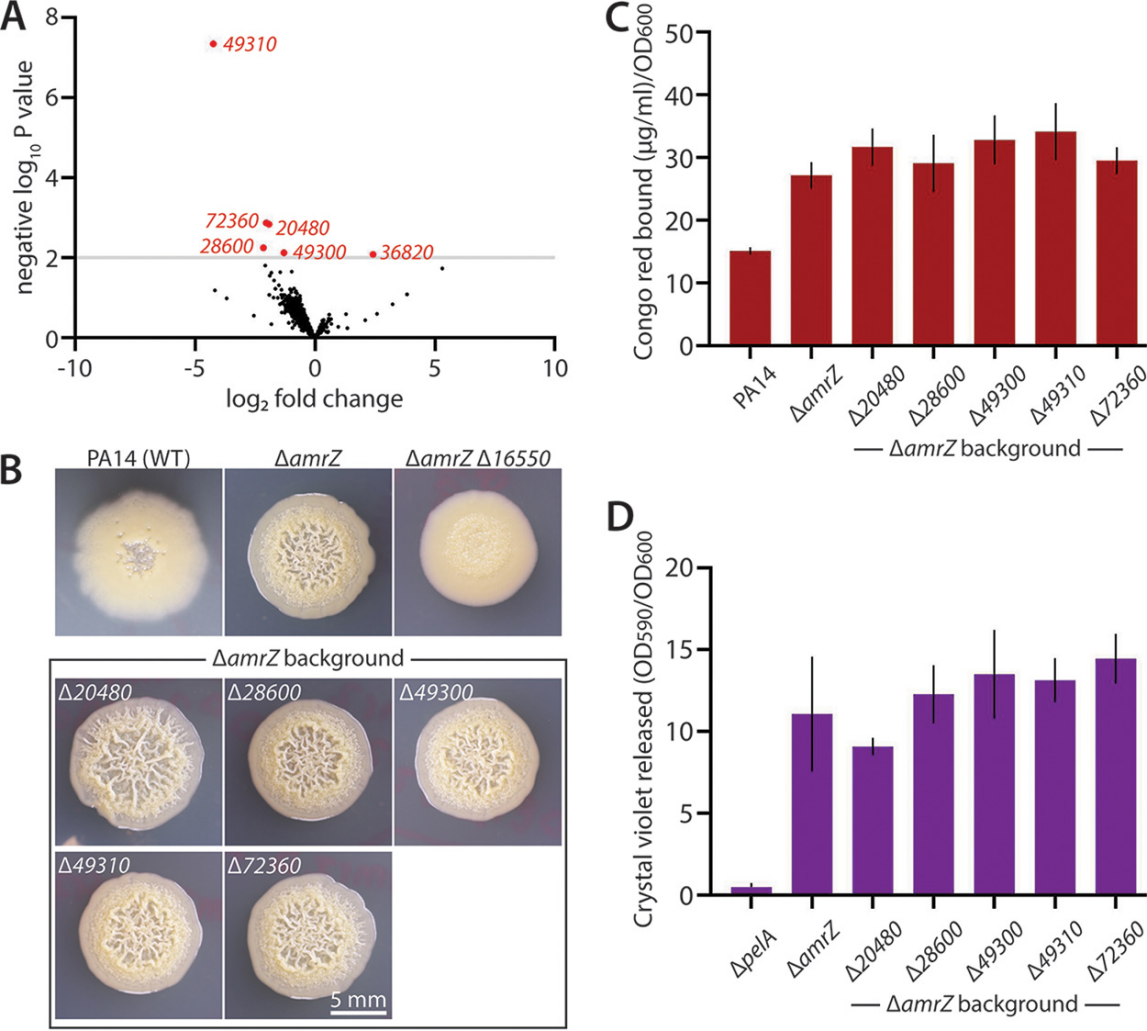
Transcriptional effects of *16550* deletion and biofilm analysis of downregulated genes. (A) Volcano plot of differentially regulated genes in a Δ*amrZ* Δ*16550* strain versus the Δ*amrZ* parent. A *P* value of 10^−2^ was set as the significance threshold (gray horizontal line). The 6 genes significantly differentially regulated in the absence of *16550* are shown in red. (B) Representative photographs of colony morphology after 6 days of growth at 25°C on M6301 agar of reference strains as noted and strains deleted for the 5 downregulated genes. (C) Congo red binding (normalized to OD_600_) by the indicated mutant strains. Mean values of at least three replicates are shown; error bars indicate ±1 standard deviation. (D) Crystal violet binding by the indicated mutants after 48 h of static growth at 37°C in M63 liquid medium. Mean values of at least three replicates are shown; error bars indicate ±1 standard deviation.

Given the impact of *16550* deletion on biofilm colony morphology (9), and because 16550 is a known DNA-binding protein (36), we hypothesized that deletion of *16550* might elicit transcriptional changes that corresponded with decreased biofilm formation. To investigate the transcriptional impact of *16550* deletion on cells growing under laboratory biofilm colony-producing conditions, we performed transcriptomic analysis on RNA isolated from P. aeruginosa colonies grown on M6301 agar. We compared the transcriptome of the moderately hyper-biofilm-forming Δ*amrZ* strain (9, 34) to its biofilm-impaired Δ*amrZ* Δ*16550* derivative grown under identical conditions. We used the Δ*amrZ* strain background because of the strong impact of *16550* deletion on Pel production in this background and for consistency with our previous work (9). We observed that deletion of *16550* had significant effects on the expression of six genes (Table 1, Fig. 1A). Among these, five genes, namely, *PA14*_*20480*, *PA14*_*28600*, *PA14*_*49300*, *PA14*_*49310*, and *PA14*_*72360*, were downregulated, whereas *PA14*_*36820* was significantly upregulated. Hence, the absence of the 16550 protein, which corresponded with a marked reduction in biofilm formation, also corresponded with a specific transcriptional impact in biofilm-grown colonies.

### Individual deletions of downregulated genes in Δ*16550* cells minimally impact biofilm phenotypes.

To test whether the genes most strongly regulated by 16550 impact biofilm formation under our laboratory conditions, we constructed in-frame deletions of the genes identified in our transcriptomic data set. We first considered whether the decrease in biofilm formation in the Δ*amrZ*Δ*16550* background was due to downregulation of one or more of the five downregulated genes we identified (Fig. 1A). If so, deletion of these genes (i.e., downregulation to zero) might, like the *16550* deletion, impact biofilm formation associated with Δ*amrZ* cells. Individual deletions of *20480*, *28600*, *49300*, *49310* (which is cotranscribed with *49300*), or *72360* in the Δ*amrZ* background showed little to no visual effect on the wrinkled morphology of Δ*amrZ* (Fig. 1B). Likewise, these deletions decreased neither Pel levels in colony biofilms as quantified by Congo red (Fig. 1C) nor the mass of surface-attached biofilms in static liquid cultures as quantified via crystal violet staining (Fig. 1D). For the sake of completeness, we also constructed the same deletions in the wild-type PA14 background, which does not form wrinkled colonies, to assess their impact (see Fig. S1 in the supplemental material). Deletion of *20480*, *28600*, *49300*, and *49310* showed little to no effect on wild-type (smooth) colony morphology (Fig. S1A and B), Pel levels (Fig. S1C and D), or surface-attached biofilm mass crystal violet staining (Fig. S1E). However, deletion of *72360* caused a dramatic increase in colony wrinkling that was accompanied by significantly increased Pel levels (Fig. S1C). Though at present it remains unclear how the effect of *72360* deletion relates to the decreased biofilm phenotype of Δ*16550* strains, the positive impact of *72360* deletion on wild-type biofilm formation at least constitutes evidence that 16550-regulated genes influence biofilm phenotypes. Overall, we conclude that none of these genes, individually at least, is responsible for decreased biofilm formation by Δ*16550* cells.

### Combinatorial deletions of downregulated genes in Δ*16550* cells minimally impact biofilm phenotypes.

**FIG 2:**
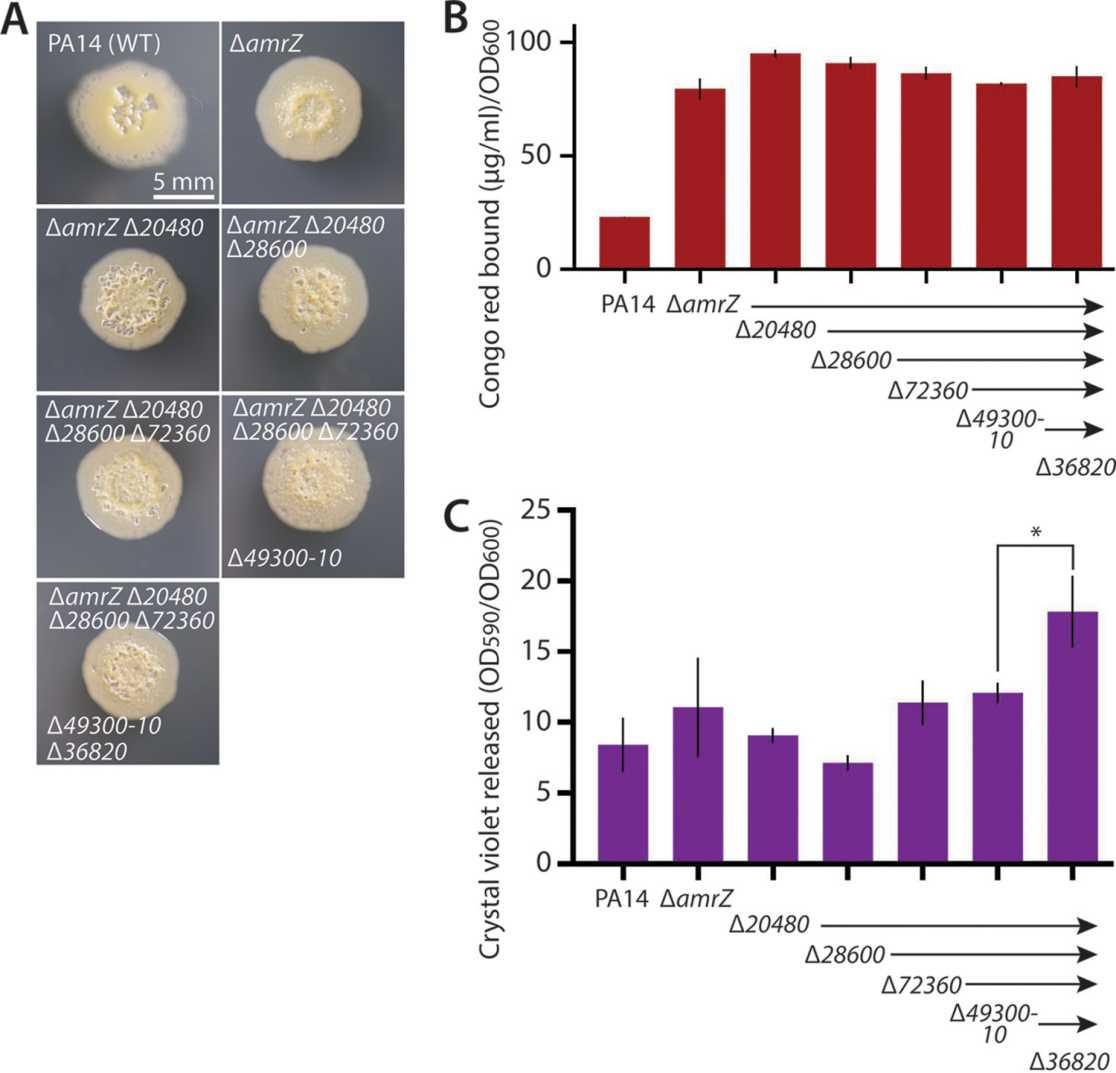
Assessing the impact of combinatorial deletions of downregulated genes on biofilm formation. (A) Representative photographs of colony morphology after 6 days of growth at 25°C on M6301 agar of reference strains as noted and strains deleted for the noted genes. (B) Congo red binding (normalized to OD_600_) by the indicated mutant strains. Mean values of at least three replicates are shown; error bars indicate ±1 standard deviation. (C) Crystal violet binding by the indicated mutants after 48 h of static growth at 37°C in M63 liquid medium. Mean values of at least three replicates are shown; error bars indicate ±1 standard deviation. *, *P* = 0.01.

We next considered the possibility that two or more of the downregulated genes may act in concert to decrease biofilm matrix levels. If so, deleting two or more of these genes together might have a greater phenotypic impact. Beginning with a Δ*amrZ* strain, we sequentially added in-frame deletions of each of the downregulated genes. Neither a strain with all five downregulated genes deleted nor any of the intermediate strains with fewer deletions had a visually discernible effect on colony morphology (Fig. 2A). Accordingly, these strains also showed no salient alterations in Pel matrix levels (Fig. 2B) or attached biofilm mass (Fig. 2C). Having generated the quintuple mutant lacking all of the downregulated genes, we additionally deleted the sole upregulated gene, *36820*, to test its impact on biofilm phenotypes. While we did not observe any changes to colony morphology (Fig. 2A) or Pel levels (Fig. 2B), *36820* deletion in the quintuple-mutant background had a modest but significant positive effect on surface-attached biofilm mass (Fig. 2C), hinting at a possible negative impact of the *36820* gene product on biofilm formation.

### *36820*-associated phenotypes are consistent with a role in negative regulation of biofilm formation.

**FIG 3:**
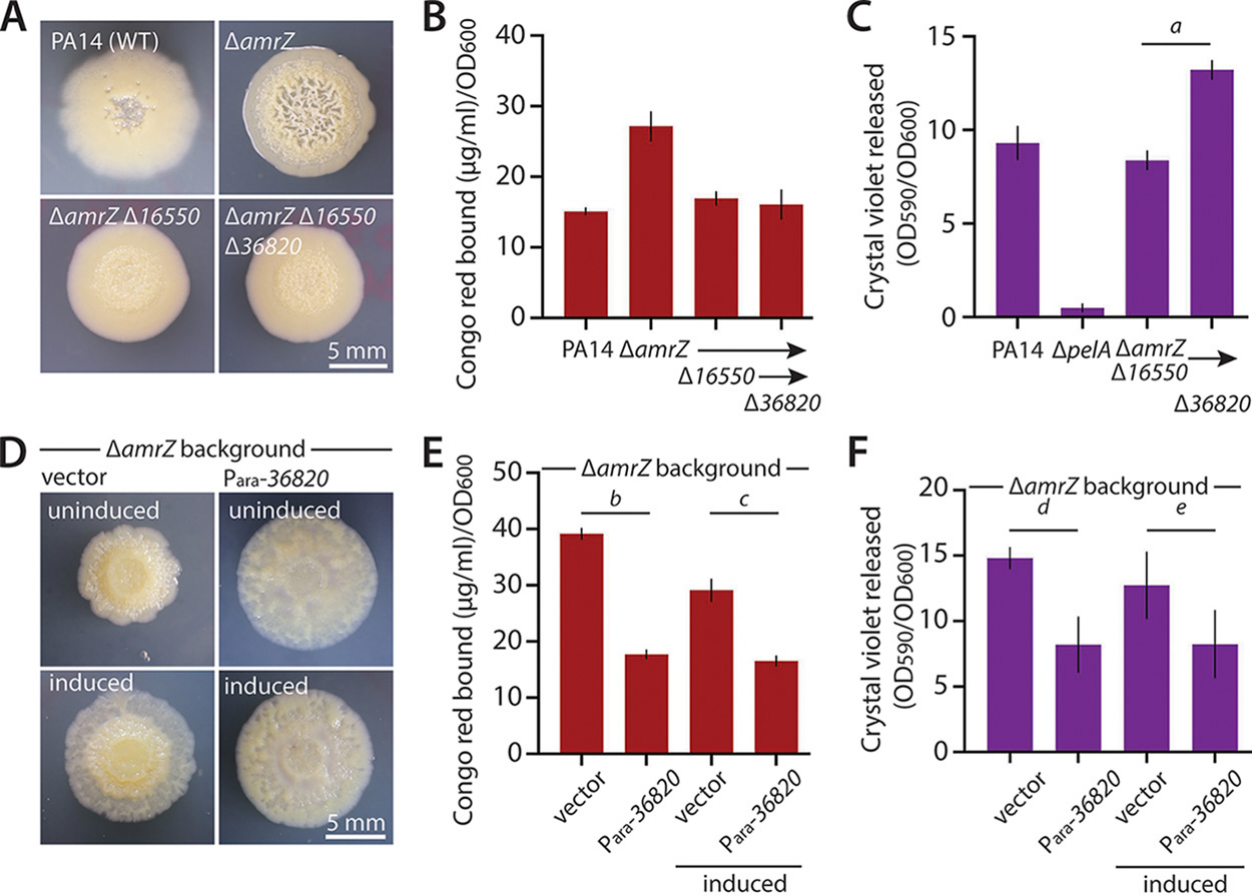
Functional analysis of *36820* deletion and overexpression on biofilm formation. (A) Representative photographs of colony morphology after 6 days of growth at 25°C on M6301 agar of strains as indicated. (B) Congo red binding (normalized to OD_600_) by the indicated mutant strains. Mean values of at least three replicates are shown; error bars indicate ±1 standard deviation. (C) Crystal violet binding by the indicated mutants after 48 h of static growth at 37°C in M63 liquid medium. Mean values of at least three replicates are shown; error bars indicate ±1 standard deviation. *a*, *P* = 0.01. (D) Representative photographs of colony morphology after 6 days of growth at 25°C on M6301 agar of Δ*amrZ* bearing empty vector pJN105 or pJN105-*36820* uninduced or induced with 1% arabinose, as indicated. (E) Congo red binding by the indicated mutant strains as in panel B. *b*, *P* = 1.1 × 10^−5^; *c*, *P* = 6.5 × 10^−4^. (F) Crystal violet binding by the indicated mutant strains as in panel C. *d*, *P* = 0.002; *e*, *P* = 0.04. Note that the PA14, Δ*amrZ*, and Δ*amrZ* Δ*16550* colonies in panel A are identical to those in Fig. 1B, as these are representative of common controls in one large experiment shown across multiple figures.

A negative impact of *36820* on biofilm formation would be consistent with its status as the only upregulated gene in the Δ*amrZ* Δ*16550* strain (Fig. 1A). If greater expression of *36820* were responsible for decreasing biofilm formation in Δ*amrZ* Δ*16550*, we would expect that deletion of *36820* might restore wrinkled morphology to Δ*amrZ* Δ*16550* cells. However, deletion of *36820* in this strain background did not restore colony wrinkling (Fig. 3A) or increase Pel levels as judged by Congo red binding (Fig. 3B), but the deletion did significantly increase attached biofilm mass (Fig. 3C). As in the quintuple-mutant background (Fig. 2B and C), this result was consistent with *36820* deletion having a greater impact on surface-attached biofilms than on agar-grown colony biofilms.

As a further test of 36820 as a potential negative regulator of biofilm formation, we overexpressed *36820* in the biofilm-forming Δ*amrZ* background using an arabinose-inducible pJN105 plasmid. In alignment with a negative role for 36820, and even without arabinose induction, strains bearing pJN105-*36820* displayed a smooth colony morphology relative to Δ*amrZ* with the empty vector (Fig. 3D). Consistent with this visual phenotype, Pel levels were significantly decreased in the presence of plasmid-borne *36820* (Fig. 3E), as was attached biofilm mass (Fig. 3F), suggesting that 36820 is indeed a negative regulator of biofilm formation. Such a role for 36820 is also supported by other data. For example, in our own transcriptomic comparison of wild-type and Δ*amrZ* strains, *36820* stood out as one of the most downregulated genes in Δ*amrZ* (Fig. S2). Hence, restoration of *36820* expression in an Δ*amrZ* Δ*16550* may at least partially explain the reduction in biofilm phenotype when *16550* is deleted.

### Deletion of genes differentially regulated in Δ*16550* cells impacts motility.

**FIG 4:**
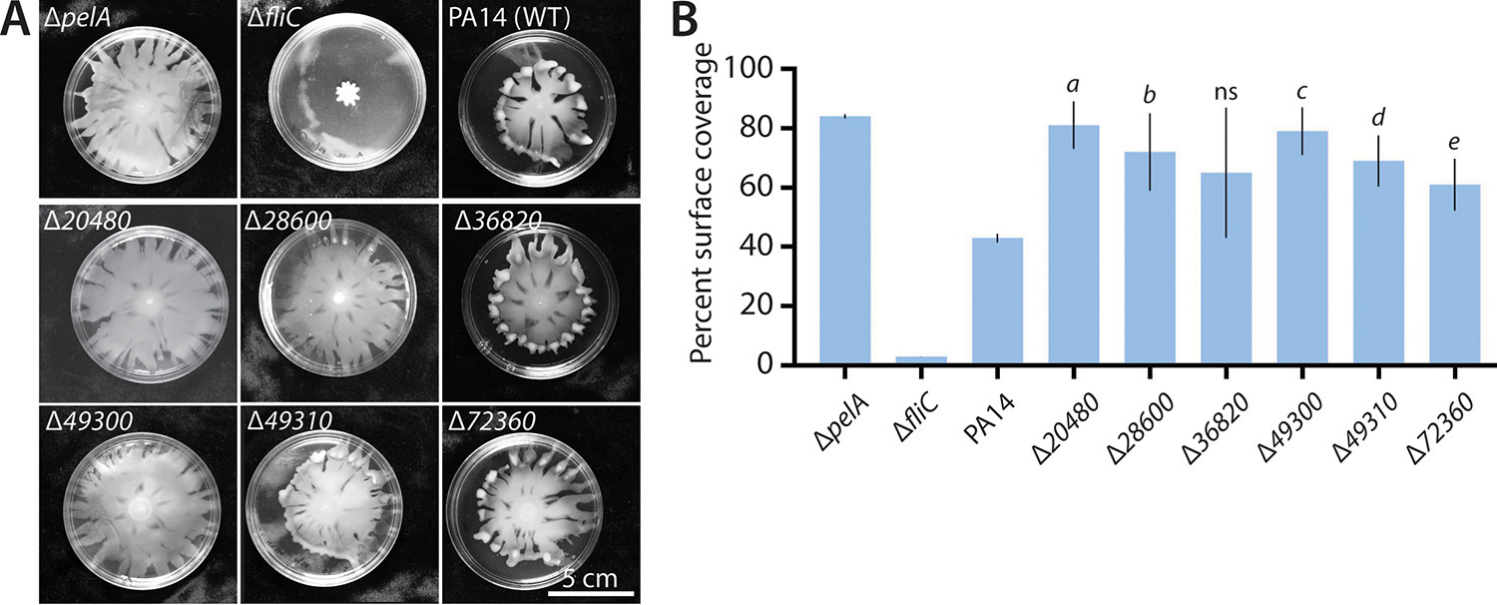
Impact of 16550-regulated gene deletion on swimming. (A) Representative photographs of swimming motility of strains (on 0.3% LB agar) deleted for the indicated genes. (B) Percent surface coverage of each indicated deletion strain. Mean values of at least three replicates are shown; error bars indicate ±1 standard deviation. *a*, *P* = 0.0013; *b*, *P* = 0.01; *c*, *P* = 0.001; *d*, *P* = 0.006; *e*, *P* = 0.02; ns, not significant (*P* > 0.05).

As a final phenotypic test of strains deleted for 16550-regulated genes, we examined motility, as the inverse relationship between biofilm formation and motility is well studied (37, 38). We tested the ability of the generated mutants to swim in 0.3% agar (Fig. 4A). As controls, we used a hypermotile (and biofilm-defective) (38) Δ*pelA* strain and a motility-defective (39) Δ*fliC* strain. Deletion of any of the five Δ*16550*-downregulated genes in a wild-type background significantly increased cell motility (as assessed by plate coverage) (Fig. 4A and B), which was at least consistent with decreased biofilm. A notable exception was Δ*36820*, concordant with a role in negatively regulating biofilm formation, as its deletion would be expected to abet biofilm formation rather than enhancing motility. Collectively, our battery of phenotypic tests of strains with manipulations to 16550-regulated genes suggested a negative biofilm regulatory role for *36820* and individually weak but potentially positive biofilm regulatory roles for the remaining genes.

### A screen for biofilm restoration uncovered RecA.

**FIG 5:**
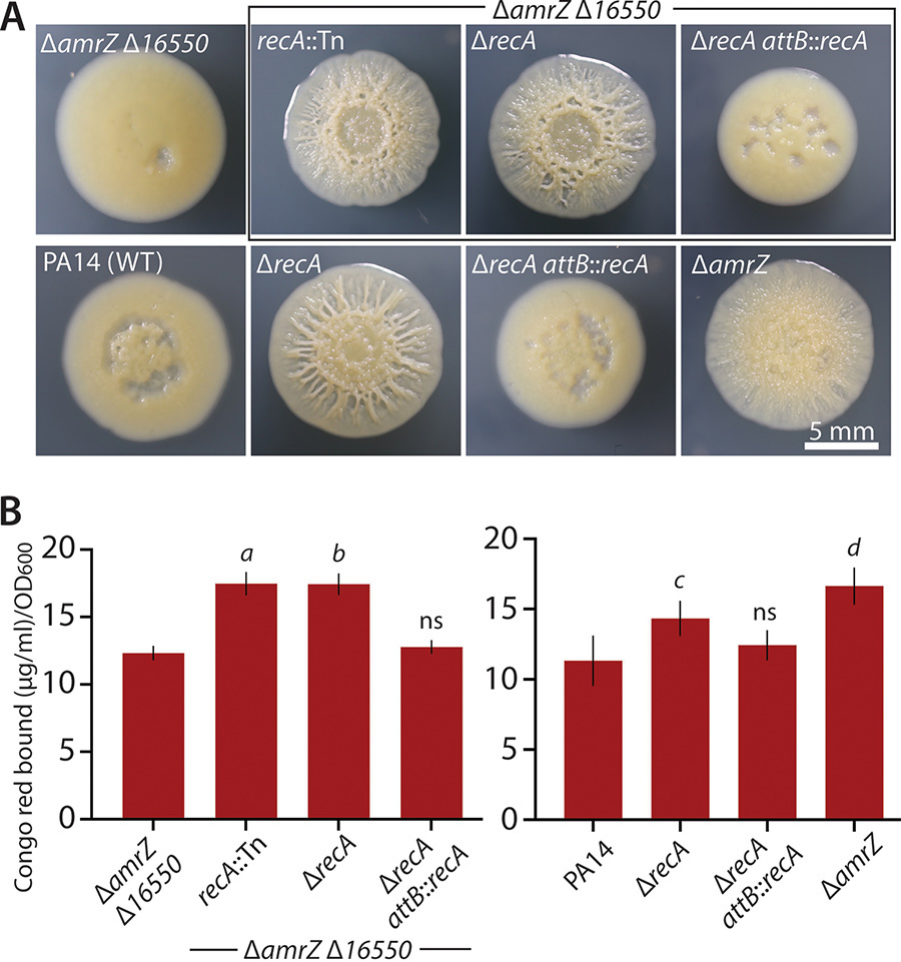
Effects of *recA* interruption or deletion on biofilm. (A) Representative photographs of colony morphology of the indicated strains after 6 days of growth at 25°C on M6301 agar. The wrinkled Δ*amrZ* strain at the bottom right is shown for comparison. (B) Congo red binding by the same strains as shown in panel A, as indicated. Error bars show standard deviations for five replicates. *P* values (Student's *t* test) are indicated by footnotes: *a*, *P* = 3.3 × 10^−6^; *b*, *P* = 2.4 × 10^−6^ (versus Δ*amrZ* Δ*16550*); *c*, *P*= 0.015; *d*, *P* = 0.0007 (versus PA14); ns, not significant (*P* > 0.05).

Besides analyzing candidate 16550-regulated genes from transcriptomic data, we additionally took a second approach to identify genes that could reverse biofilm suppression in a Δ*amrZ* Δ*16550* strain. We initiated a transposon mutagenesis screen to identify genes that, when inactivated in a Δ*amrZ* Δ*16550* parental strain, would reverse the smooth colony morphology associated with *16550* deletion, restoring wrinkled colony morphology. Of approximately 3,000 transposon mutants, we recovered approximately 95 mutants with enhanced colony wrinkling, representing a hit rate of just under 3%. Surprisingly, this screen uncovered *recA*, encoding the broadly conserved RecA recombination protein. Transposon insertion in *recA* produced a wrinkled colony morphology in the Δ*amrZ* Δ*16550* strain (Fig. 5A), with significantly higher Pel abundance than in the parental strain (Fig. 5B). An in-frame markerless deletion of *recA* in the same parental background (Δ*amrZ* Δ*16550*) showed the same restoration of colony wrinkling (Fig. 5A), and complementation of the *recA* gene at the neutral *attB* locus resulted in a smooth colony (Fig. 5A), indicating that *recA* is responsible for the biofilm phenotype. We then asked whether the biofilm-enhancing effect of *recA* deletion was specific to the Δ*amrZ* Δ*16550* background. When we deleted *recA* in the wild-type PA14 background, which normally displays smooth colony morphology in our colony morphology assay, we observed significant enhancement of colony wrinkling (Fig. 5A) and likewise elevated Pel levels (Fig. 5B). This result implied that the effect of *recA* deletion is not specific to Δ*16550* strains, but rather that *recA* deletion is more generally associated with enhanced biofilm formation. In agreement with RecA being unconnected with 16550, the expression levels of *recA* in our transcriptomic data were not significantly influenced by deletion of *16550* (downregulated 1.38-fold; *P* = 0.23). Notably, we also recovered a transposon insertion in *recF* with increased colony wrinkling, and an in-frame deletion of *recF* likewise showed enhanced colony wrinkling across wild-type, Δ*amrZ*, and Δ*amrZ* Δ*16550* strain backgrounds (Fig. S3). Our identification of RecF bolstered the notion that the loss of recombination and repair-related proteins like RecA and RecF can impact biofilm formation.

### Disabling homologous recombination increases biofilm levels.

**FIG 6:**
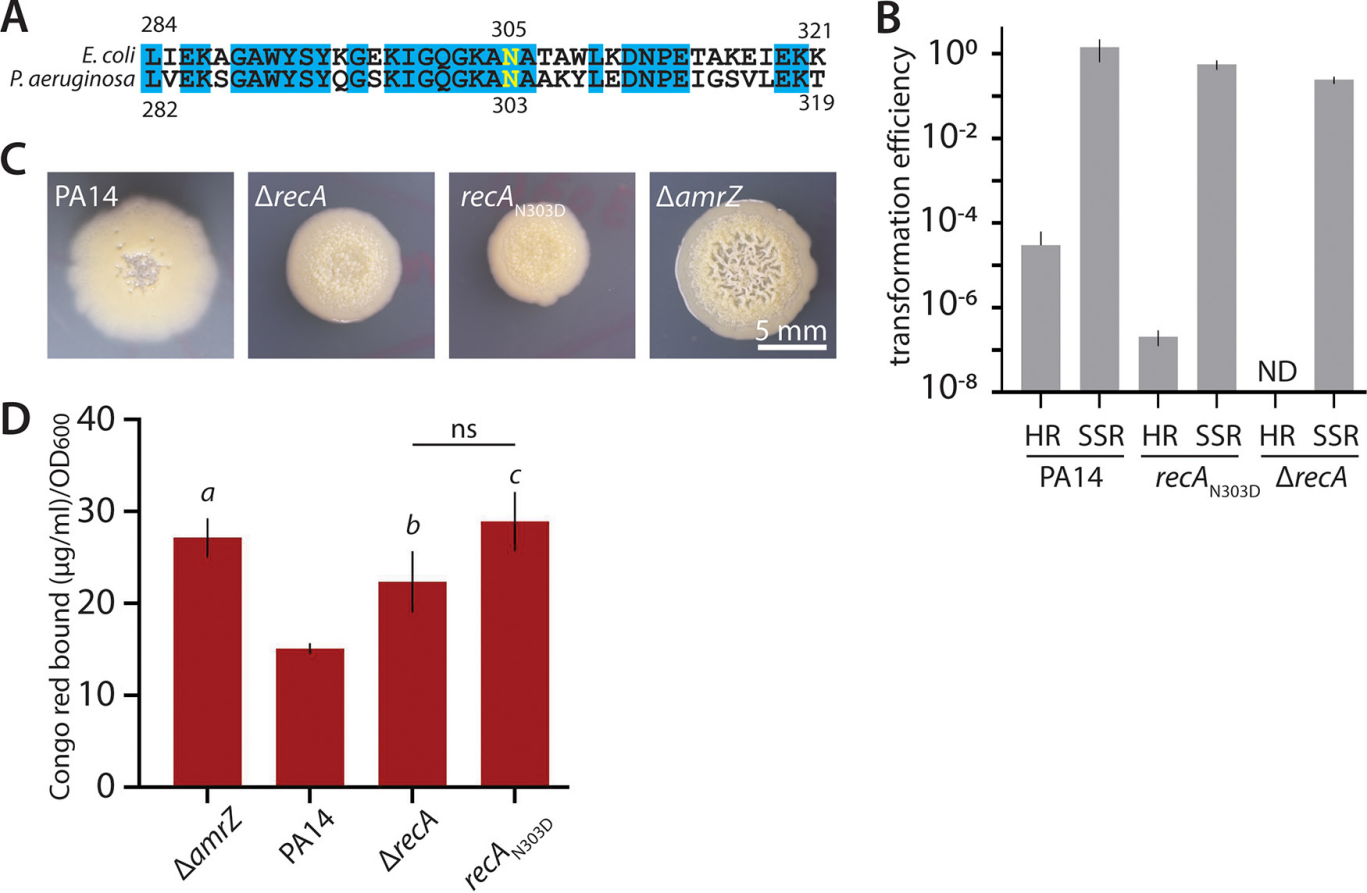
Effect of disabling RecA-mediated homologous recombination on biofilm formation. (A) Alignment of the C-terminal portion of RecA sequences from E. coli K-12 and P. aeruginosa PA14. Conservation is indicated by cyan shading, and the mutated asparagine is illustrated in yellow. (B) Transformation efficiency of the indicated strains. HR, transformation requiring homologous recombination (pEXG2); SSR, transformation via site-specific recombination (pCTX-1); ND, not detected. Error bars show standard deviations for three biological replicates. Error bars appear asymmetrical due to the log scale of the *y* axis; no lower error bar appears for PA14 because it would be taller than the bar. (C) Representative photographs of colony morphology after 6 days of growth at 25°C on M6301 agar of the indicated strains. The wrinkled Δ*amrZ* strain is shown for comparison. (D) Congo red binding by the same strains as in panel C, as indicated. Error bars show standard deviations for five replicates. *P* values (Student's *t* test) versus PA14: *a*, *P* = 0.00067 *b*, *P* = 0.02; *c*, *P* = 0.001; ns, not significant (*P* > 0.05, Δ*recA* versus *recA*_N303D_). Note that the PA14 and Δ*amrZ* colonies in panel C are identical to those in Fig. 1B and 3A, as these are representative of common controls in one large experiment shown across multiple figures.

RecA is a ubiquitous protein that is present in most bacteria and plays a key role in two cell processes, namely, homologous recombination (HR) and the DNA damage response (SOS response) (40, 41). During HR, RecA promotes strand exchange by promoting invasion of homologous double-stranded DNA (41). Upon DNA damage, RecA activates the SOS response by functioning as a coprotease to promote cleavage of LexA, a transcriptional repressor that normally holds the SOS response in an off state. Because RecA has two distinct functions, we wondered whether loss of one or both of these functions was associated with elevated biofilm production. We first turned our attention to the HR function of RecA. To specifically inactivate its HR function, we constructed a version of *recA* encoding a previously described amino acid substitution (with an Asn-to-Asp change at position 303; RecA_N303D_) (40). This substitution was used in E. coli to specifically disable HR while not interfering with its role in the SOS response, as a strain bearing *recA_N303D_* was not as sensitive to UV irradiation as SOS mutants were (40). Because RecA_N303D_ has not to our knowledge been reported in P. aeruginosa, we constructed strains containing an allelic replacement, substituting *recA_N303D_* for the wild-type gene at the native locus (Fig. 6A). We assessed loss of HR by measuring transformation efficiency with a nonreplicative plasmid, pEXG2, containing a 1.2-kb region of homology with the PA14 chromosome at the *ptsN* locus (chosen for being unrelated to this study), which requires HR for integration. As a control for the efficiency of the mating process, we used a different nonreplicative plasmid, pCTX-1, which integrates via site-specific recombination. As we expected, strains carrying *recA_N303D_* displayed markedly impaired integration of pEXG2 (efficiencies of 1.18 × 10^−7^ to 3.17 × 10^−7^, compared to 0.1 × 10^−5^ to 7.8 × 10^−5^ for the wild type), whereas integration of pCTX-1 was unaffected (Fig. 6B). Importantly, the *recA*_N303D_ strain was less sensitive to UV than the *recA* deletion (Fig. S4A), indicating that SOS activation was not abolished. To test the role of HR in the enhanced-biofilm phenotype seen in Δ*amrZ* Δ*16550* Δ*recA* (Fig. 5A), we examined the colony morphology of *recA_N303D_* strains in both the wild-type (Fig. 6C) and Δ*amrZ* Δ*16550* (Fig. S4B) backgrounds. In both cases, the HR-disabled strains showed colony wrinkling (Fig. 6C, Fig. S4B) and Pel levels (Fig. 6D, Fig. S4C) that were visibly enhanced relative to levels in their *recA*^+^ counterparts. These data suggested an association between disabled HR and the enhanced biofilm phenotype of Δ*recA* strains.

### Disabling the SOS response increases biofilm levels.

**FIG 7:**
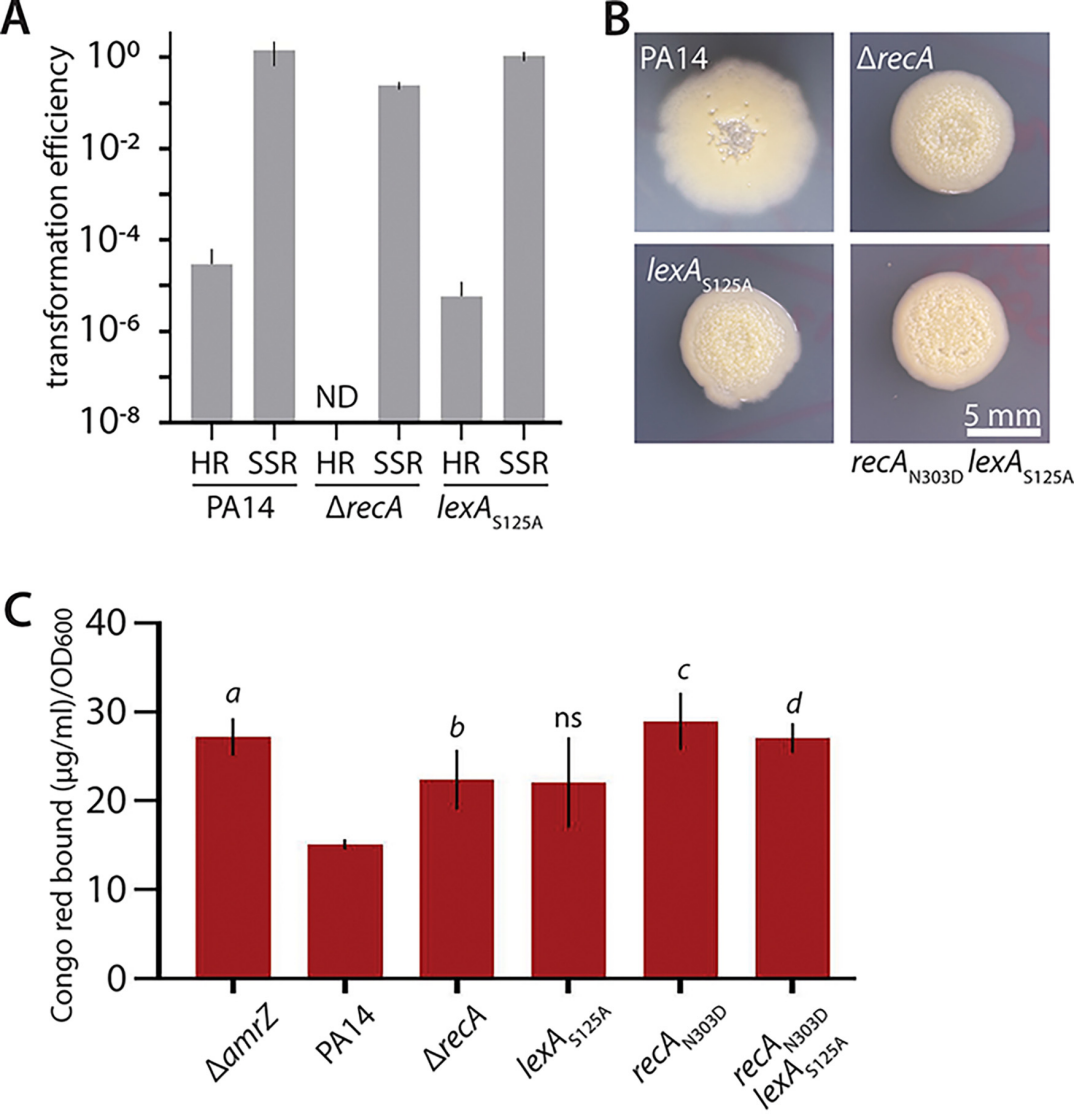
Effect of disabling SOS signaling alone or in combination with homologous recombination on biofilm formation. (A) Transformation efficiency of the indicated strains. HR, transformation requiring homologous recombination (pEXG2); SSR, transformation via site-specific recombination (pCTX-1); ND, not detected. Error bars show standard deviations for three biological replicates. (B) Representative photographs of colony morphology after 6 days of growth at 25°C on M6301 agar of the indicated strains. (C) Congo red binding by the indicated strains. The data for PA14 Δ*amrZ*, Δ*recA*, and *recA*_N303D_ are identical to those shown in Fig. 6D. Error bars show standard deviations for five replicates. *P* values (Student's *t* test) versus PA14: *a*, *P* = 0.00067; *b*, *P* = 0.02; *c*, *P* = 0.001; *d*, *P* = 0.0002; ns, not significant (*P* = 0.07). Note that the PA14 colony in panel B is identical to those in Fig. 1B, 3A, and 6C and that the Δ*recA* colony is identical to that in Fig. 6C, as these are representative of common controls in one large experiment shown across multiple figures.

We then examined the impact of the second function of RecA, in activating the SOS response, on biofilm formation. To specifically disable the SOS response, we constructed a previously reported version of *lexA* encoding an amino acid substitution that disables cleavage of LexA (LexA_S125A_) (42). A *lexA_S125A_* mutant in P. aeruginosa had been previously reported to be hypersensitive to UV irradiation and was used to define the SOS regulon in this species (42). We confirmed that this mutation did not substantially affect HR (Fig. 7A), but it did affect UV sensitivity (Fig. S4A). We then examined the colony morphology of *lexA_S125A_* strains in the wild-type (Fig. 7B) and Δ*amrZ* Δ*16550* (Fig. S4B and C) strain backgrounds. As in HR-disabled strains, SOS-disabled strains showed greater colony wrinkling (Fig. 7B, Fig. S4B) and greater Pel abundance (Fig. 7C, Fig. S4C) than *lexA*^+^ strains, with similar effects to the full *recA* deletion. To confirm that both functions are important for the increase in biofilm formation in Δ*recA*, we introduced both point mutations in the same background (PA14 *lexA_S125A_ recA_N303D_*). The double mutant displayed increased colony wrinkling (Fig. 7B) and Pel matrix levels, but combining both mutations did not have an additive effect on Pel levels (Fig. 7C). Collectively, our results argued that loss of either function of RecA, HR or SOS activation, can contribute to the increased biofilm formation observed in strains lacking *recA*.

### Deletion of RecA increases biofilm formation independently of cellular c-di-GMP levels.

**FIG 8:**
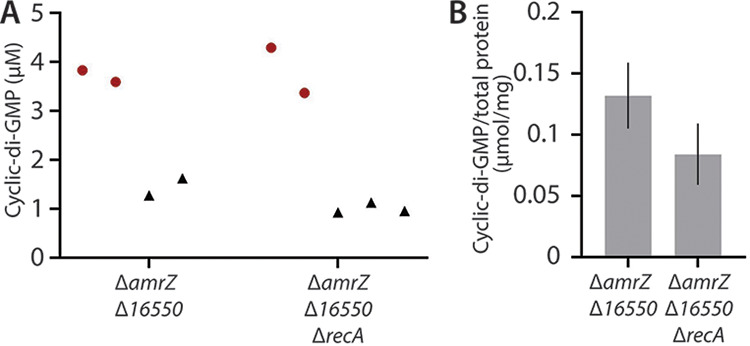
Impact of *recA* deletion on cellular cyclic-di-GMP levels. (A) Cyclic-di-GMP concentrations in extracts from the indicated strains (not normalized to cellular protein levels). Each symbol represents a separate experiment. Black triangles are extracts from 2 OD units of cells; red circles are extracts from 10 OD units of cells. Cyclic-di-GMP was quantified by HPLC in comparison to known standards. (B) Cyclic-di-GMP levels in extracts from 10 OD units of cells from the indicated strains (corresponding to the red circles in panel A) normalized to total cellular protein. Error bars show standard deviations of two replicates.

Because c-di-GMP is an important second messenger in biofilm formation, we finally asked whether deletion of *recA* impacted c-di-GMP levels. Deletion of *16550* from a Δ*amrZ* strain was previously reported to result in substantially lower c-di-GMP levels (9). Is the increased colony wrinkling and matrix production in Δ*amrZ* Δ*16550* Δ*recA* (Fig. 5A and B) associated with increased c-di-GMP? Interestingly, deletion of *recA* did not have any substantial effect on c-di-GMP levels relative to those in Δ*amrZ* Δ*16550*, with the Δ*recA* strain trending toward lower [c-di-GMP] (Fig. 8). The fact that both Δ*amrZ* Δ*16550* Δ*recA* and Δ*amrZ* Δ*16550* exhibited similar c-di-GMP levels but had distinct phenotypes suggested that the biofilm effects of *recA* deletion may be mediated via pathways other than c-di-GMP signaling.

## DISCUSSION

In this study, transcriptomic analysis revealed that deletion of *16550* significantly changed the expression of only a small set of six genes (Table 1). Constructing individual deletions of these genes and assessing the colony morphology of the resulting strains to test their potential impact on biofilm formation suggested that two of these genes (primarily *36820* but also *72360*) appear to have an individual impact on biofilm formation. Interestingly, we also found that the genes each impacted motility in a manner consistent with their putative roles in biofilm formation, namely, *36820* negatively impacted biofilm formation and the remaining genes positively impacted biofilm formation. The strongest role we identified was for *36820*, as its ectopic expression lowered biofilm levels (Fig. 3D to F), and its deletion increased biofilm formation under some conditions (Fig. 2C and 3C). Moreover, *36820* was strongly downregulated when *amrZ* was deleted (Fig. S2A and B). Curiously, and in contrast to our findings, its ortholog in PAO1, *PA2146*, has been characterized as a gene that is required for biofilm formation and resistance to tobramycin, as its inactivation suppresses biofilm formation and renders biofilms susceptible to tobramycin (43). This discrepancy may be due to variations between PAO1 and PA14 or different methods of biofilm formation (colonies versus continuous flow reactors). In any case, the relatively small set of genes regulated by 16550 is consistent with 16550 controlling specific aspects of P. aeruginosa physiology, and further exploring other functions of these genes, especially *36820*, will be an interesting topic for future work.

**TABLE 1:** Differentially regulated genes in Δ*amrZ* Δ*16550* versus Δ*amrZ*

Gene	Log_2_ fold change	*P* value	Notes on putative functions and/or regulation
PA14_*49310*	−4.23	4.5 × 10^−8^	Repressed by LasR (65)
PA14_*72360*	−2.03	0.001	Repressed by LasR and RhlR (65); its deletion impaired biofilm (66)
PA14_*20480*	−1.9	0.001	Repressed by LasR and RhlR (65); activated by MvfR (65)
PA14_*28600*	−2.15	0.005	Repressed by LasR and RhlR (65); activated by MvfR (65)
PA14_*49300*	−1.29	0.007	Lipoxygenase
PA14_*36820*	2.42	0.009	Annotated as general stress protein; deletion impairs biofilm formation and resistance to tobramycin (43); activated by AmrZ in PAO1 (34, 67) and highly downregulated in Δ*amrZ* relative to wild type (67-fold-downregulated in Δ*amrZ*; *P* = 6.38 × 10^−10^)

We also found here that reversal of biofilm suppression in a Δ*16550* strain could be achieved by disruption of genes that were not under *16550* control. Using a mutagenesis and visual screening approach (9) to find genes whose deletion could reverse biofilm suppression in a Δ*16550* strain, we unexpectedly identified the recombination protein-encoding gene *recA*. Because RecA functions both in homologous recombination and in the SOS response, we genetically dissected these functions and separately assessed their contributions to biofilm phenotypes. Our data argued that both functions of RecA contribute to the increased biofilm formation observed in a *recA* deletion mutant. How deletion of *recA* results in increased biofilm formation remains unanswered, although our data suggest that it does not involve alterations in c-di-GMP levels. Moreover, because RecA has two distinct functions and disabling either of these functions separately increased biofilm formation, loss of each function may have different downstream effects.

One possibility is that LexA may repress a gene involved in biofilm suppression. In this scenario, deletion of *recA* or the presence of LexA_S125A_ would cause LexA to constitutively repress that gene, making Δ*recA* or *lexA*_S125A_ strains tend toward biofilm production. Similar logic appears to be present in Acinetobacter baumannii, where deletion of *recA* increases biofilm formation via *bfmR*, a global biofilm regulator, through a protein called UmuDAb. While A. baumannii lacks LexA (44–46), UmuDab is a known repressor (47, 48) that appears unique to this species (49). However, unlike the repressor function of LexA, Ching et al. proposed that UmuDab might be an activator of *bfmR* (44).

Consistent with the colony wrinkling associated with deletion of *recA*, we also identified a transposon insertion in *recF* in our morphology screen as having increased colony wrinkling (Fig. S3). RecF, along with other proteins, is a recombination mediator protein that facilitates RecA loading on gapped DNA substrates, leading to the formation of the RecA nucleoprotein filament and its subsequent activation (50–52). Deletion of *recF* in the Δ*amrZ* Δ*16550* and wild-type backgrounds caused a similar enhancement to colony wrinkling as did deletion of *recA* (Fig. S3). We speculate that impairment of RecA loading and subsequent activity in a *recF* mutant is the cause of its elevated biofilm formation.

High intracellular levels of c-di-GMP are typically associated with biofilm formation (33). However, our data suggest that deletion of *recA* increases biofilm formation independently of cellular c-di-GMP levels, as its deletion did not have any apparent effect on c-di-GMP levels relative to Δ*amrZ* Δ*16550*. One possible interpretation of this result is that deletion of *recA* causes changes to a local c-di-GMP pool while leaving overall cellular c-di-GMP levels unchanged, as has been previously reported in certain other cases (53). It is more likely that the biofilm effects of *recA* deletion might be mediated via a presently unknown pathway that does not involve c-di-GMP signaling.

The relationship between RecA and biofilm formation does not appear to be uniform across different bacterial organisms. Deletion of *recA* increases biofilm formation in E. coli (54). Conversely, a RecA-deficient mutant produced lower-density biofilms in Streptococcus mutans (55). Gotoh et al. reported that deletion of *recA* or disabling cleavage of LexA (LexA_S125A_) did not increase biofilm mass production in comparison to wild type in P. aeruginosa PAO1 (56), a finding echoed by Chellappa et al. (57). A possible explanation for this difference is the different EPS produced by these common model strains. PAO1 produces an additional EPS, Psl, which has an important role in PAO1 biofilm formation, whereas PA14 does not produce Psl and instead relies only on Pel (26, 58, 59). Collectively, it seems that deletion of *recA* does not have a unanimous effect on biofilm formation. Further study is required to elucidate the mechanisms by which deficiencies in RecA function can stimulate biofilm formation in P. aeruginosa PA14.

## MATERIALS AND METHODS

### Strains and growth conditions.

P. aeruginosa PA14 and E. coli SM10 were grown in Luria Bertani (LB)-Lennox broth (10 g/liter tryptone, 5 g/liter yeast extract, 5 g/liter NaCl) with shaking or on solid medium (fortified with 1.5% Bacto agar) at 37°C. To select for antibiotic markers, 75 μg/mL or 20 μg/mL gentamicin or 25 μg/mL tetracycline was added to media as appropriate. P. aeruginosa was selected after mating with E. coli by addition of 25 μg/mL irgasan to media or by use of Vogel-Bonner minimal medium (VBMM), which contains citrate as its sole carbon source and does not support growth of E. coli (60). The strains used in this study are listed in Table 2 and Table S1 in the supplemental material. All mutations in P. aeruginosa were markerless in-frame deletions or point mutations generated using allelic replacement with the pEXG2 vector, with counterselection on LB plates containing 6% sucrose or on no-salt LB plates containing 15% sucrose (60). All mutations were screened by diagnostic PCR (deletions) or sequencing (point mutations). Gene complementation was conducted using mini-CTX-1 integrants at the chromosomal *attB* locus. Plasmids and primers used in this study are listed in Tables S2 and S3, respectively, and modes of strain construction are listed in Text S1.

**TABLE 2:** P. aeruginosa strains used in this study

Strain	Genotype or description	Source or reference
PA14 (MTC1)	Laboratory wild-type strain of P. aeruginosa	Laboratory stock; Stephen Lory, Harvard Medical School
CSS12	PA14 Δ*amrZ*	9
CSS995	PA14 Δ*amrZ* Δ*20480*	This study
CSS978	PA14 Δ*amrZ* Δ*28600*	This study
CSS959	PA14 Δ*amrZ* Δ*49300*	This study
CSS741	PA14 Δ*amrZ* Δ*49310*	This study
CSS1518	PA14 Δ*amrZ* Δ*72360*	This study
CSS997	PA14 Δ*20480*	This study
CSS1604	PA14 Δ*28600*	This study
CSS818	PA14 Δ*36820*	This study
CSS976	PA14 Δ*49300*	This study
CSS740	PA14 Δ*49310*	This study
CSS1517	PA14 Δ*72360*	This study
CSS1673	PA14 Δ*amrZ* Δ*20480* Δ*28600*	This study
CSS1705	PA14 Δ*amrZ* Δ*20480* Δ*28600* Δ*72360*	This study
CSS1716	PA14 Δ*amrZ* Δ*20480* Δ*28600* Δ*72360* Δ*49300* Δ*49310*	This study
CSS1747	PA14 Δ*amrZ* Δ*20480* Δ*28600* Δ*72360* Δ*49300* Δ*49310* Δ*36820*	This study
MTC1381	PA14 Δ*amrZ* Δ*16550*	9
MTC798	PA14 Δ*pelA*	This study
CSS824	PA14 Δ*amrZ* Δ*36820*	This study
CSS825	PA14 Δ*amrZ* Δ*16550* Δ*36820*	This study
CSS1472	PA14 Δ*amrZ* pJN105	This study
CSS1473	PA14 Δ*amrZ* pJN105-*36820*	This study
MTC1134	PA14 Δ*fliC*	This study
CSS310	PA14 Δ*amrZ* Δ*16550 recA*:: Tn	This study
CSS788	PA14 Δ*recA*	This study
CSS790	PA14 Δ*amrZ* Δ*16550*Δ*recA*	This study
CSS1108	PA14 Δ*recA attB*::CTX-1-*recA*	This study
CSS1110	PA14 Δ*amrZ* Δ*16550* Δ*recA attB*::CTX-1-*recA*	This study
CSS260	PA14 Δ*amrZ* Δ*16550 recF*:: Tn	This study
CSS632	PA14 Δ*recF*	This study
CSS633	PA14 Δ*amrZ* Δ*recF*	This study
CSS664	PA14 Δ*amrZ* Δ*16550* Δ*recF*	This study
CSS1202	PA14 *recA_N303D_*	This study
CSS985	PA14 Δ*amrZ* Δ*16550 recA_N303D_*	This study
CSS1315	PA14 *lexA_S125A_*	This study
CSS987	PA14 Δ*amrZ* Δ*16550 lexA_S125A_*	This study
CSS1746	PA14 *lexA_S125A_ recA_N303D_*	This study

### Transposon mutagenesis and biofilm colony morphology screen.

Transposon mutagenesis and biofilm colony morphology screening were performed as previously described (9). In brief, P. aeruginosa PA14 Δ*amrZ*Δ*16550* (MTC1381) was mated with *E. coli* SM10 pBT24 (MTC33). We then screened for transposon mutants that had restored colony wrinkling as an indication of biofilm formation.

### Biofilm assay.

Colony morphology of P. aeruginosa strains was assessed on M6301 agar medium containing 1% agar, 100 μM KH_2_PO_4_, 15.14 mM (NH_4_)_2_SO_4_, and 0.36 μM FeSO_4_·H_2_O (pH balanced to 7.0 using 10 M KOH) (61). After autoclaving, 1 mM MgSO_4_, 0.5% glycerol, and 0.2% Casamino Acids (BD Bacto, USA) were added. M6301 agar was made fresh before each experiment; 40 mL was poured in each plate and allowed to solidify overnight. P. aeruginosa cultures were grown overnight with shaking in 3 mL liquid LB at 37°C and then normalized to an optical density at 600 nm (OD_600_) of 1. Droplets (2 μL) of the normalized cultures were spotted onto the hardened M6301 agar plates. The plates were incubated right side up at 25°C and were photographed after 6 days.

For polysaccharide content estimation, colonies were collected on day 6 from the agar and homogenized for at least 20 s with an Argos rotary pestle (Cole-Parmer, USA) in 1 mL of sterile phosphate-buffered saline (PBS). After allowing flakes generated from resuspending the colonies to settle, 100 μL was removed from the resuspension to measure the OD_600_ using a Biotek Synergy HT plate reader. The remaining suspension was pelleted using a microcentrifuge at 13,000 × *g* for 3 min at room temperature. After discarding the supernatant, the pellets were resuspended in 40 μg mL^−1^ Congo red and agitated on a GyroMixer XL (GeneMate, VWR) for 90 min at room temperature. The samples were then centrifuged again at 13,000 × *g* for 3 min at room temperature, and the OD of the supernatants was measured at 490 nm. A standard curve was generated by measuring the OD_490_ of Congo red at 40, 20, 10, 5, 2, 1, and 0.5 μg mL^−1^. PBS (1×) was used as a blank and as the diluent for the standard curve.

To maximize consistency across the data presented in this study, many of the colony morphology and Congo red binding data shown were taken from the same large experiment that had common controls. Hence, the colony morphology and Congo red binding data for strain PA14 are repeated in Fig. 1B, 3A, 6C, and 7B; Δ*amrZ* data are repeated in Fig. 1B, 3A, and 6C; Δ*amrZ* Δ*16550* data are repeated in Fig. 1B and 3A; and Δ*recA* data appear in Fig. 6C and 7B.

### Crystal violet biofilm formation assay.

The crystal violet biofilm formation assay was conducted as previously described (62). In brief, strains were grown overnight in LB medium. The following day, their OD_600_ was measured, and cells were normalized to an OD_600_ of 0.1 and incubated in M630 medium at 37°C for 48 h. After incubating the strains for 48 h, their OD_600_ was measured, and unattached cells were removed from the wells by decanting. A 96-well plate was then submerged in a small tub of deionized water 3 to 4 times and shaken to remove any excess water. Two hundred microliters of a 0.1% solution of crystal violet in water was used to stain each well in the 96-well plate. After staining the cells at room temperature for 15 min, the plate was rinsed 3 to 4 times with deionized water by submerging the plate in a small tub of water (the water was changed between washes). The plate was then dried for 20 to 30 min, and 30% acetic acid in water was added to each well to solubilize the crystal violet. To quantify the biofilm, the absorbance was measured in a plate reader at 550 nm using 30% acetic acid in water as the blank.

### Motility assay.

Swimming motility was assessed as previously described (63). In brief, strains were inoculated at the center of LB plates with 0.3% agar and incubated overnight at 37°C. To determine the percent surface coverage, the plate was photographed with a Canon EOS digital camera. The image was converted to grayscale and the brightness and contrast were adjusted using Photoshop software (Adobe, Mountain View, CA) to provide appropriate contrast between the agar surface and the bacterial colony. The colony was specifically selected using the “magic wand” tool in Photoshop, and the number of pixels contained in the selection (i.e., proportional to the area of the colony) was divided by the number of pixels in the entire plate (i.e., the area of the plate).

### UV light sensitivity assay.

Strains were grown overnight in liquid LB at 37°C. The following day, the overnight-grown cultures were normalized to an OD_600_ of 1 and were serially diluted. Then, 10 μL was spotted on a plain LB agar plate and exposed to 10 μJ of UV light (approximately 10 s) using a Stratalinker UV cross-linker 2400 (Stratagene, La Jolla, CA).

### Quantification of intracellular cyclic di-GMP.

P. aeruginosa PA14 and derivative strains were grown overnight in 3 mL LB liquid shaking culture at 37°C. Cultures were further spotted on M6301 agar as described above. After 3 days of growth at 25°C, colonies were collected for cyclic di-GMP extraction. Using a sterile spatula, the colonies were collected and homogenized with 1 mL of sterile PBS in 1.5-mL microcentrifuge tubes for at least 20 s with an Argos rotary pestle. Cyclic di-GMP was extracted from either 2 or 10 OD units of cells (1 OD unit = 1 mL of cell suspension at an OD_600_ of 1.0) and quantified by high-performance liquid chromatography (HPLC) as previously described (64) with a few modifications. The HPLC gradient was modified to the following: 0 to 2 min, 1% solvent B; 7 min, 10% B; 12 min, 15% B; 17 min, 18% B; 22 min, 20% B; 27 min, 22% B; 32 min, 24% B; 37 min, 26% B. This gradient resulted in the elution of c-di-GMP at approximately 6.4 to 6.5 min. The concentration of c-di-GMP in each sample was calculated from a standard curve prepared using commercially available pure c-di-GMP. In some cases, the c-di-GMP concentration in the extract was normalized to the total amount of protein from the same sample (calculated using the Bradford method with bovine serum albumin standards).

### Transformation efficiency assay.

The transformation efficiency of P. aeruginosa PA14 and derivative strains was assessed by mating with E. coli SM10 containing pEXG2 bearing approximately 1.2 kb of homology to the *ptsN* locus, whose integration into the host chromosome requires homologous recombination. P. aeruginosa PA14 and Escherichia coli SM10 pEXG2-*ptsN* were mated as previously described (60). In brief, 0.5 mL of the P. aeruginosa recipient strain and 1.5 mL of the E. coli donor strain were placed in separate 2-mL microcentrifuge tubes and centrifuged at 10,000 × *g* for 5 min at room temperature. After discarding the supernatant, each pellet was resuspended in 50 μL of LB. Both 50-μL cell mixtures were then combined and placed on a prewarmed LB agar plate and incubated overnight at 30°C. Using a sterile spatula, the mating matrix was collected and resuspended in 1 mL of LB. The OD_600_ of the mating matrix of all the strains tested was normalized to an OD_600_ of 10 and further serially diluted. Dilutions (100 μL) were spread on LB-agar plates and incubated overnight at 37°C before manual enumeration of CFU.

### RNA isolation and sequencing.

Total RNA was isolated from homogenized colonies grown in quadruplicate on M6301–1% agar plates for 3 days at 25°C using the New England Biolabs Monarch total RNA miniprep kit. Quality-control steps, rRNA depletion, Illumina library preparation, and 150-bp paired-end high-throughput Illumina sequencing were performed by Novogene (Beijing, China). Sequence mapping and analysis were performed at the Oklahoma University Health Sciences Center Laboratory for Molecular Biology and Cytometry Research using CLC software. Complete lists of differentially regulated genes for PA14 versus Δ*amrZ* and for Δ*amrZ* versus Δ*amrZ* Δ*16550* comparisons are provided in Data Set S1.

### Statistical comparisons.

All pairwise comparisons were statistically analyzed using two-tailed Student’s *t* tests, assuming equal variance.

### Data availability.

The full transcriptomic data sets, including the raw sequence reads, have been deposited in the Gene Expression Omnibus at NCBI under accession number GSE226104.

## References

[1] Mulcahy LR, Isabella VM, Lewis K. 2014. Pseudomonas aeruginosa biofilms in disease. Microb Ecol 68:1–12. doi:10.1007/s00248-013-0297-x. 24096885 PMC3977026

[2] Bahador N, Shoja S, Faridi F, Dozandeh-Mobarrez B, Qeshmi FI, Javadpour S, Mokhtary S. 2019. Molecular detection of virulence factors and biofilm formation in Pseudomonas aeruginosa obtained from different clinical specimens in Bandar Abbas. Iran J Microbiol 11:25–30. 30996828 PMC6462266

[3] Stewart PS. 2002. Mechanisms of antibiotic resistance in bacterial biofilms. Int J Med Microbiol 292:107–113. doi:10.1078/1438-4221-00196. 12195733

[4] Vishwakarma V. 2020. Impact of environmental biofilms: industrial components and its remediation. J Basic Microbiol 60:198–206. doi:10.1002/jobm.201900569. 31856349

[5] Khatoon Z, McTiernan CD, Suuronen EJ, Mah T-F, Alarcon EI. 2018. Bacterial biofilm formation on implantable devices and approaches to its treatment and prevention. Heliyon 4:e01067. doi:10.1016/j.heliyon.2018.e01067. 30619958 PMC6312881

[6] Irie Y, Starkey M, Edwards AN, Wozniak DJ, Romeo T, Parsek MR. 2010. Pseudomonas aeruginosa biofilm matrix polysaccharide Psl is regulated transcriptionally by RpoS and post-transcriptionally by RsmA. Mol Microbiol 78:158–172. 20735777 10.1111/j.1365-2958.2010.07320.x PMC2984543

[7] Romero D, Aguilar C, Losick R, Kolter R. 2010. Amyloid fibers provide structural integrity to Bacillus subtilis biofilms. Proc Natl Acad Sci USA 107:2230–2234. doi:10.1073/pnas.0910560107. 20080671 PMC2836674

[8] Bokranz W, Wang X, Tschäpe H, Römling U. 2005. Expression of cellulose and curli fimbriae by Escherichia coli isolated from the gastrointestinal tract. J Med Microbiol 54:1171–1182. doi:10.1099/jmm.0.46064-0. 16278431

[9] Cabeen MT, Leiman SA, Losick R. 2016. Colony-morphology screening uncovers a role for the Pseudomonas aeruginosa nitrogen-related phosphotransferase system in biofilm formation. Mol Microbiol 99:557–570. doi:10.1111/mmi.13250. 26483285 PMC5130288

[10] Caiazza NC, O'Toole GA. 2004. SadB is required for the transition from reversible to irreversible attachment during biofilm formation by Pseudomonas aeruginosa PA14. J Bacteriol 186:4476–4485. doi:10.1128/JB.186.14.4476-4485.2004. 15231779 PMC438627

[11] Cowles KN, Gitai Z. 2010. Surface association and the MreB cytoskeleton regulate pilus production, localization and function in Pseudomonas aeruginosa. Mol Microbiol 76:1411–1426. doi:10.1111/j.1365-2958.2010.07132.x. 20398206 PMC3132575

[12] Guvener ZT, Harwood CS. 2007. Subcellular location characteristics of the Pseudomonas aeruginosa GGDEF protein, WspR, indicate that it produces cyclic-di-GMP in response to growth on surfaces. Mol Microbiol 66:1459–1473. 18028314 10.1111/j.1365-2958.2007.06008.x PMC4105145

[13] Irie Y, Borlee BR, O’Connor JR, Hill PJ, Harwood CS, Wozniak DJ, Parsek MR. 2012. Self-produced exopolysaccharide is a signal that stimulates biofilm formation in Pseudomonas aeruginosa. Proc Natl Acad Sci USA 109:20632–20636. doi:10.1073/pnas.1217993109. 23175784 PMC3528562

[14] O'Toole GA, Kolter R. 1998. Flagellar and twitching motility are necessary for Pseudomonas aeruginosa biofilm development. Mol Microbiol 30:295–304. doi:10.1046/j.1365-2958.1998.01062.x. 9791175

[15] Montie TC, Doyle-Huntzinger D, Craven RC, Holder IA. 1982. Loss of virulence associated with absence of flagellum in an isogenic mutant of Pseudomonas aeruginosa in the burned-mouse model. Infect Immun 38:1296–1298. doi:10.1128/iai.38.3.1296-1298.1982. 6818148 PMC347889

[16] De Weger LA, van der Lugt CI, Wijfjes AH, Bakker PA, Schippers B, Lugtenberg B. 1987. Flagella of a plant-growth-stimulating Pseudomonas fluorescens strain are required for colonization of potato roots. J Bacteriol 169:2769–2773. doi:10.1128/jb.169.6.2769-2773.1987. 3294806 PMC212183

[17] Grant CC, Konkel ME, Cieplak W, Jr, Tompkins LS. 1993. Role of flagella in adherence, internalization, and translocation of Campylobacter jejuni in nonpolarized and polarized epithelial cell cultures. Infect Immun 61:1764–1771. doi:10.1128/iai.61.5.1764-1771.1993. 8478066 PMC280763

[18] Korber DR, Lawrence JR, Caldwell DE. 1994. Effect of motility on surface colonization and reproductive success of Pseudomonas fluorescens in dual-dilution continuous culture and batch culture systems. Appl Environ Microbiol 60:1421–1429. doi:10.1128/aem.60.5.1421-1429.1994. 16349247 PMC201498

[19] Simpson DA, Ramphal R, Lory S. 1995. Characterization of Pseudomonas aeruginosa fliO, a gene involved in flagellar biosynthesis and adherence. Infect Immun 63:2950–2957. doi:10.1128/iai.63.8.2950-2957.1995. 7622217 PMC173401

[20] Tian M, Wu Z, Zhang R, Yuan J. 2022. A new mode of swimming in singly flagellated Pseudomonas aeruginosa. Proc Natl Acad Sci USA 119:e2120508119. doi:10.1073/pnas.2120508119. 35349348 PMC9168846

[21] Kazmierczak BI, Schniederberend M, Jain R. 2015. Cross-regulation of Pseudomonas motility systems: the intimate relationship between flagella, pili and virulence. Curr Opin Microbiol 28:78–82. doi:10.1016/j.mib.2015.07.017. 26476804 PMC4688086

[22] Colvin KM, Irie U, Tart CS, Urbano R, Whitney JC, Ryder C, Howell PL, Wozniak DJ, Parsek MR. 2012. The Pel and Psl polysaccharides provide Pseudomonas aeruginosa structural redundancy within the biofilm matrix. Environ Microbiol 14:1913–1928. doi:10.1111/j.1462-2920.2011.02657.x. 22176658 PMC3840794

[23] Marmont LS, Whitfield GB, Rich JD, Yip P, Giesbrecht LB, Stremick CA, Whitney JC, Parsek MR, Harrison JJ, Howell PL. 2017. PelA and PelB proteins form a modification and secretion complex essential for Pel polysaccharide-dependent biofilm formation in Pseudomonas aeruginosa. J Biol Chem 292:19411–19422. doi:10.1074/jbc.M117.812842. 28972168 PMC5702679

[24] Whitfield GB, Marmont LS, Howell PL. 2015. Enzymatic modifications of exopolysaccharides enhance bacterial persistence. Front Microbiol 6:471. doi:10.3389/fmicb.2015.00471. 26029200 PMC4432689

[25] Friedman L, Kolter R. 2004. Genes involved in matrix formation in Pseudomonas aeruginosa PA14 biofilms. Mol Microbiol 51:675–690. doi:10.1046/j.1365-2958.2003.03877.x. 14731271

[26] Colvin KM, Gordon VD, Murakami K, Borlee BR, Wozniak DJ, Wong GCL, Parsek MR. 2011. The pel polysaccharide can serve a structural and protective role in the biofilm matrix of Pseudomonas aeruginosa. PLoS Pathog 7:e1001264. doi:10.1371/journal.ppat.1001264. 21298031 PMC3029257

[27] Flemming HC, Wingender J. 2010. The biofilm matrix. Nat Rev Microbiol 8:623–633. doi:10.1038/nrmicro2415. 20676145

[28] Matsuyama BY, Krasteva PV, Baraquet C, Harwood CS, Sondermann H, Navarro MVAS. 2016. Mechanistic insights into c-di-GMP-dependent control of the biofilm regulator FleQ from Pseudomonas aeruginosa. Proc Natl Acad Sci USA 113:E209–E218. doi:10.1073/pnas.1523148113. 26712005 PMC4720306

[29] Hickman JW, Harwood CS. 2008. Identification of FleQ from Pseudomonas aeruginosa as a c-di-GMP-responsive transcription factor. Mol Microbiol 69:376–389. doi:10.1111/j.1365-2958.2008.06281.x. 18485075 PMC2612001

[30] Lee VT, Matewish JM, Kessler JL, Hyodo M, Hayakawa Y, Lory S. 2007. A cyclic-di-GMP receptor required for bacterial exopolysaccharide production. Mol Microbiol 65:1474–1484. doi:10.1111/j.1365-2958.2007.05879.x. 17824927 PMC2170427

[31] Simm R, Morr M, Kader A, Nimtz M, Römling U. 2004. GGDEF and EAL domains inversely regulate cyclic di-GMP levels and transition from sessility to motility. Mol Microbiol 53:1123–1134. doi:10.1111/j.1365-2958.2004.04206.x. 15306016

[32] Whitney JC, Colvin KM, Marmont LS, Robinson H, Parsek MR, Howell PL. 2012. Structure of the cytoplasmic region of PelD, a degenerate diguanylate cyclase receptor that regulates exopolysaccharide production in Pseudomonas aeruginosa. J Biol Chem 287:23582–23593. doi:10.1074/jbc.M112.375378. 22605337 PMC3390633

[33] Hengge R. 2009. Principles of c-di-GMP signalling in bacteria. Nat Rev Microbiol 7:263–273. doi:10.1038/nrmicro2109. 19287449

[34] Jones CJ, Newsom D, Kelly B, Irie Y, Jennings LK, Xu B, Limoli DH, Harrison JJ, Parsek MR, White P, Wozniak DJ. 2014. ChIP-Seq and RNA-Seq reveal an AmrZ-mediated mechanism for cyclic di-GMP synthesis and biofilm development by Pseudomonas aeruginosa. PLoS Pathog 10:e1003984. doi:10.1371/journal.ppat.1003984. 24603766 PMC3946381

[35] Hou L, Debru A, Chen Q, Bao Q, Li K. 2019. AmrZ regulates swarming motility through cyclic di-GMP-dependent motility inhibition and controlling Pel polysaccharide production in Pseudomonas aeruginosa PA14. Front Microbiol 10:1847. doi:10.3389/fmicb.2019.01847. 31474950 PMC6707383

[36] Longo F, Rampioni G, Bondi R, Imperi F, Fimia GM, Visca P, Zennaro E, Leoni L. 2013. A new transcriptional repressor of the Pseudomonas aeruginosa quorum sensing receptor gene lasR. PLoS One 8:e69554. doi:10.1371/journal.pone.0069554. 23861975 PMC3702619

[37] Kuchma SL, Brothers KM, Merritt JH, Liberati NT, Ausubel FM, O'Toole GA. 2007. BifA, a cyclic-Di-GMP phosphodiesterase, inversely regulates biofilm formation and swarming motility by Pseudomonas aeruginosa PA14. J Bacteriol 189:8165–8178. doi:10.1128/JB.00586-07. 17586641 PMC2168662

[38] Caiazza NC, Merritt JH, Brothers KM, O'Toole GA. 2007. Inverse regulation of biofilm formation and swarming motility by Pseudomonas aeruginosa PA14. J Bacteriol 189:3603–3612. doi:10.1128/JB.01685-06. 17337585 PMC1855903

[39] Murray TS, Kazmierczak BI. 2008. Pseudomonas aeruginosa exhibits sliding motility in the absence of type IV pili and flagella. J Bacteriol 190:2700–2708. doi:10.1128/JB.01620-07. 18065549 PMC2293233

[40] Adikesavan AK, Katsonis P, Marciano DC, Lua R, Herman C, Lichtare O. 2011. Separation of recombination and SOS response in Escherichia coli RecA suggests LexA interaction sites. PLoS Genet 7:e1002244. doi:10.1371/journal.pgen.1002244. 21912525 PMC3164682

[41] Michel B. 2005. After 30 years of study, the bacterial SOS response still surprises us. PLoS Biol 3:e255. doi:10.1371/journal.pbio.0030255. 16000023 PMC1174825

[42] Cirz RT, O’Neill BM, Hammond JA, Head SR, Romesberg FE. 2006. Defining the Pseudomonas aeruginosa SOS response and its role in the global response to the antibiotic ciprofloxacin. J Bacteriol 188:7101–7110. doi:10.1128/JB.00807-06. 17015649 PMC1636241

[43] Kaleta MF, Petrova OE, Zampaloni C, Garcia-Alcalde F, Parker M, Sauer K. 2022. A previously uncharacterized gene, PA2146, contributes to biofilm formation and drug tolerance across the deltaproteobacteria. NPJ Biofilms Microbiomes 8:54. doi:10.1038/s41522-022-00314-y. 35798749 PMC9262955

[44] Ching C, Muller P, Brychcy M, Reverdy A, Nguyen B, Downs M, Regan S, Isley B, Fowle W, Chai Y, Godoy VG. 2020. RecA levels modulate biofilm development in Acinetobacter baumannii. bioRxiv. doi:10.1101/809392. 37918886

[45] Ching C, Gozzi K, Heinemann B, Chai Y, Godoy VG. 2017. RNA-mediated cis regulation in Acinetobacter baumannii modulates stress-induced phenotypic variation. J Bacteriol 199. doi:10.1128/JB.00799-16. PMC5424255 28320880

[46] Robinson A, Brzoska AJ, Turner KM, Withers R, Harry EJ, Lewis PJ, Dixon NE. 2010. Essential biological processes of an emerging pathogen: DNA replication, transcription, and cell division in Acinetobacter spp. Microbiol Mol Biol Rev 74:273–297. doi:10.1128/MMBR.00048-09. 20508250 PMC2884411

[47] Hare JM, Ferrell JC, Witkowski TA, Grice AN. 2014. Prophage induction and differential RecA and UmuDAb transcriptome regulation in the DNA damage responses of Acinetobacter baumannii and Acinetobacter baylyi. PLoS One 9:e93861. doi:10.1371/journal.pone.0093861. 24709747 PMC3978071

[48] Witkowski TA, Grice AN, Stinnett DB, Wells WK, Peterson MA, Hare JM. 2016. UmuDAb: an error-prone polymerase accessory homolog whose N-terminal domain is required for repression of DNA damage inducible gene expression in Acinetobacter baylyi. PLoS One 11:e0152013. doi:10.1371/journal.pone.0152013. 27010837 PMC4807011

[49] Hare JM, Adhikari S, Lambert KV, Hare AE, Grice AN. 2012. The Acinetobacter regulatory UmuDAb protein cleaves in response to DNA damage with chimeric LexA/UmuD characteristics. FEMS Microbiol Lett 334:57–65. doi:10.1111/j.1574-6968.2012.02618.x. 22697494 PMC3475745

[50] Beernink HT, Morrical SW. 1999. RMPs: recombination/replication mediator proteins. Trends Biochem Sci 24:385–389. doi:10.1016/S0968-0004(99)01451-6. 10500302

[51] Sakai A, Cox MM. 2009. RecFOR and RecOR as distinct RecA loading pathways. J Biol Chem 284:3264–3272. doi:10.1074/jbc.M807220200. 18986990 PMC2631980

[52] Lenhart JS, Branes ER, Schroeder JW, Sorenson RJ, Showalter HD, Simmons LA. 2014. RecO and RecR are necessary for RecA loading in response to DNA damage and replication fork stress. J Bacteriol 196:2851–2860. doi:10.1128/JB.01494-14. 24891441 PMC4135682

[53] Merritt JH, Ha D-G, Cowles KN, Lu W, Morales DK, Rabinowitz J, Gitai Z, O’Toole GA. 2010. Specific control of Pseudomonas aeruginosa surface-associated behaviors by two c-di-GMP diguanylate cyclases. mBio 1. doi:10.1128/mBio.00183-10. PMC2957078 20978535

[54] Beloin C, Valle J, Latour-Lambert P, Faure P, Kzreminski M, Balestrino D, Haagensen JAJ, Molin S, Prensier G, Arbeille B, Ghigo J-M. 2004. Global impact of mature biofilm lifestyle on Escherichia coli K-12 gene expression. Mol Microbiol 51:659–674. doi:10.1046/j.1365-2958.2003.03865.x. 14731270

[55] Inagaki S, Matsumoto-Nakano M, Fujita K, Nagayama K, Funao J, Ooshima T. 2009. Effects of recombinase A deficiency on biofilm formation by Streptococcus mutans. Oral Microbiol Immunol 24:104–108. doi:10.1111/j.1399-302X.2008.00480.x. 19239636

[56] Gotoh H, Kasaraneni N, Devineni N, Dallo SF, Weitao T. 2010. SOS involvement in stress-inducible biofilm formation. Biofouling 26:603–611. doi:10.1080/08927014.2010.501895. 20603726

[57] Chellappa ST, Maredia R, Phipps K, Haskins WE, Weitao T. 2013. Motility of Pseudomonas aeruginosa contributes to SOS-inducible biofilm formation. Res Microbiol 164:1019–1027. doi:10.1016/j.resmic.2013.10.001. 24125694

[58] Yang L, Hu Y, Liu Y, Zhang J, Ulstrup J, Molin S. 2011. Distinct roles of extracellular polymeric substances in Pseudomonas aeruginosa biofilm development. Environ Microbiol 13:1705–1717. doi:10.1111/j.1462-2920.2011.02503.x. 21605307

[59] Ghafoor A, Hay ID, Rehm BH. 2011. Role of exopolysaccharides in Pseudomonas aeruginosa biofilm formation and architecture. Appl Environ Microbiol 77:5238–5246. doi:10.1128/AEM.00637-11. 21666010 PMC3147449

[60] Hmelo LR, Borlee BR, Almblad H, Love ME, Randall TE, Tseng BS, Lin C, Irie Y, Storek KM, Yang JJ, Siehnel RJ, Howell PL, Singh PK, Tolker-Nielsen T, Parsek MR, Schweizer HP, Harrison JJ. 2015. Precision-engineering the Pseudomonas aeruginosa genome with two-step allelic exchange. Nat Protoc 10:1820–1841. doi:10.1038/nprot.2015.115. 26492139 PMC4862005

[61] Cabeen MT. 2014. Stationary phase-specific virulence factor overproduction by a lasR mutant of Pseudomonas aeruginosa. PLoS One 9:e88743. doi:10.1371/journal.pone.0088743. 24533146 PMC3923063

[62] O'Toole GA. 2011. Microtiter dish biofilm formation assay. J Vis Exp 2011:2437. doi:10.3791/2437. PMC3182663 21307833

[63] Toutain CM, Zegans ME, O'Toole GA. 2005. Evidence for two flagellar stators and their role in the motility of Pseudomonas aeruginosa. J Bacteriol 187:771–777. doi:10.1128/JB.187.2.771-777.2005. 15629949 PMC543560

[64] Roy AB, Petrova OE, Sauer K. 2013. Extraction and quantification of cyclic di-GMP from P. aeruginosa. Bio Protoc 3:e828. doi:10.21769/bioprotoc.828. PMC4241849 25429368

[65] Rasmussen TB, Skindersoe ME, Bjarnsholt T, Phipps RK, Christensen KB, Jensen PO, Andersen JB, Koch B, Larsen TO, Hentzer M, Eberl L, Hoiby N, Givskov M. 2005. Identity and effects of quorum-sensing inhibitors produced by Penicillium species. Microbiology (Reading) 151:1325–1340. doi:10.1099/mic.0.27715-0. 15870443

[66] Musken M, Di Fiore S, Dotsch A, Fischer R, Haussler S. 2010. Genetic determinants of Pseudomonas aeruginosa biofilm establishment. Microbiology (Reading) 156:431–441. doi:10.1099/mic.0.033290-0. 19850623

[67] Xu B, Ju Y, Soukup RJ, Ramsey DM, Fishel R, Wysocki VH, Wozniak DJ. 2016. The Pseudomonas aeruginosa AmrZ C-terminal domain mediates tetramerization and is required for its activator and repressor functions. Environ Microbiol Rep 8:85–90. doi:10.1111/1758-2229.12354. 26549743 PMC4769699

