# Supplementary material for: Distinct Screening Approaches Uncover PA14_36820 and RecA as Negative Regulators of Biofilm Phenotypes in Pseudomonas aeruginosa PA14
Source: Microbiol Spectr. 2023 Mar 27;11(2):e03774-22. doi: 10.1128/spectrum.03774-22 (PMC10100956; doi:10.1128/spectrum.03774-22)
Supplement: Supplemental file 1 — Fig. S1 to S4, Tables S1 and S2, and supplemental text. Download spectrum.03774-22-s0001.pdf, PDF file, 0.9 MB [file spectrum.03774-22-s0001.pdf]

Supplemental Information | Yahya *et al.*

Supplemental Figures

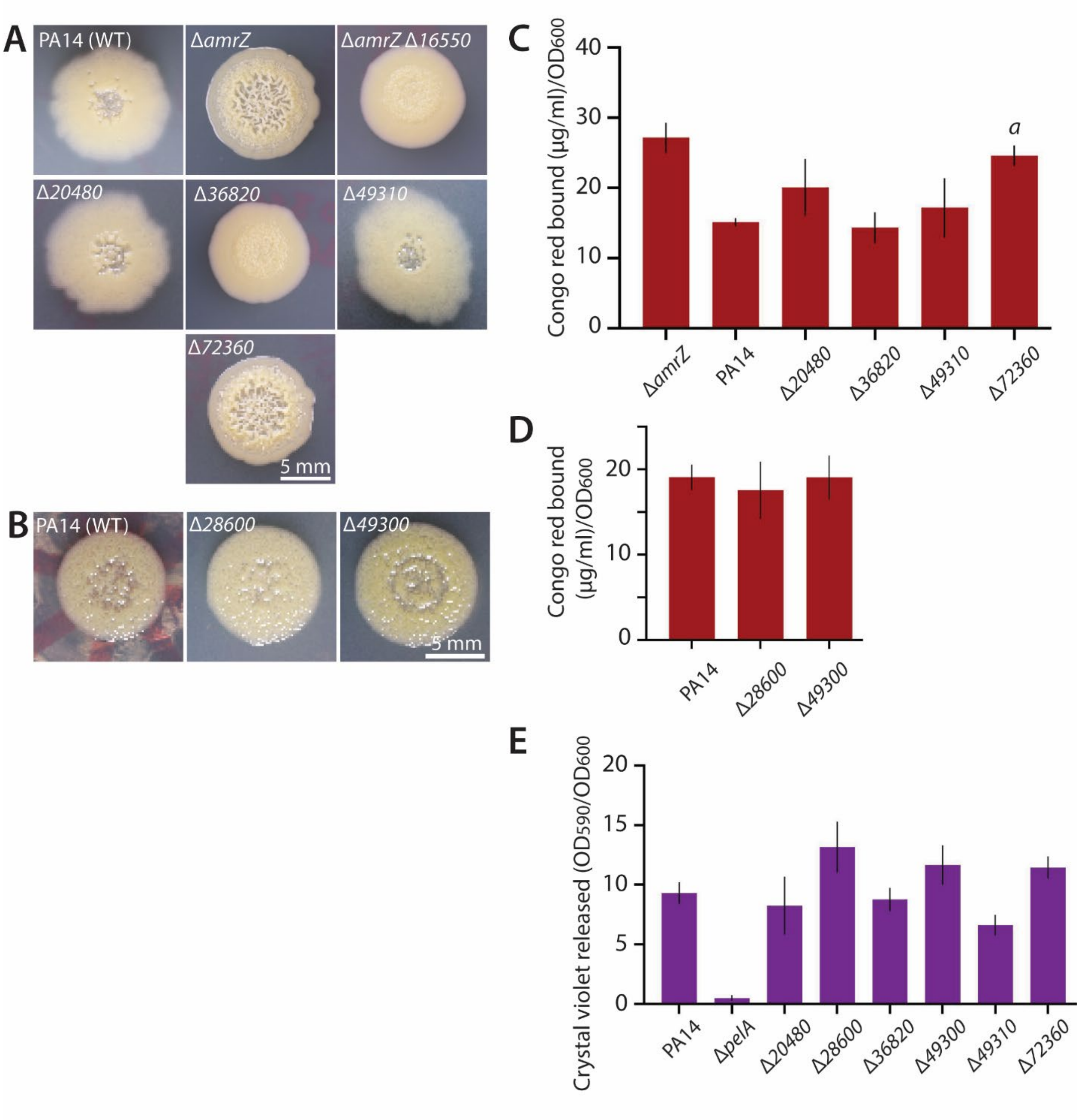

Figure S1

Figure S1. Transcriptional effects of *16550* deletion and biofilm analysis of downregulated genes. (A) Representative photographs of colony morphology after 6 d of growth at 25°C on M6301 agar of reference strains as noted and strains deleted for the 5 downregulated genes in a wild-type PA14 background. (B) Photographs as in panel A of the indicated strains (tested in a separate experiment). (C) Congo red binding (normalized to OD_{600}) by the indicated mutant strains corresponding to panel A. Mean values of at least three replicates are shown; error bars indicate ± 1 standard deviation. P-values, Student's t-test, vs. PA14: *a*, P = 0.00046. The other deletions were not significant (P > 0.05). (D) Congo red binding as in panel C of the indicated strains corresponding to panel B (tested in a separate experiment). (E) Crystal violet binding by the indicated mutants after 48 h of static growth at 37°C in M63 liquid medium. Mean values of at least three replicates are shown; error bars indicate ± 1 standard deviation.

A

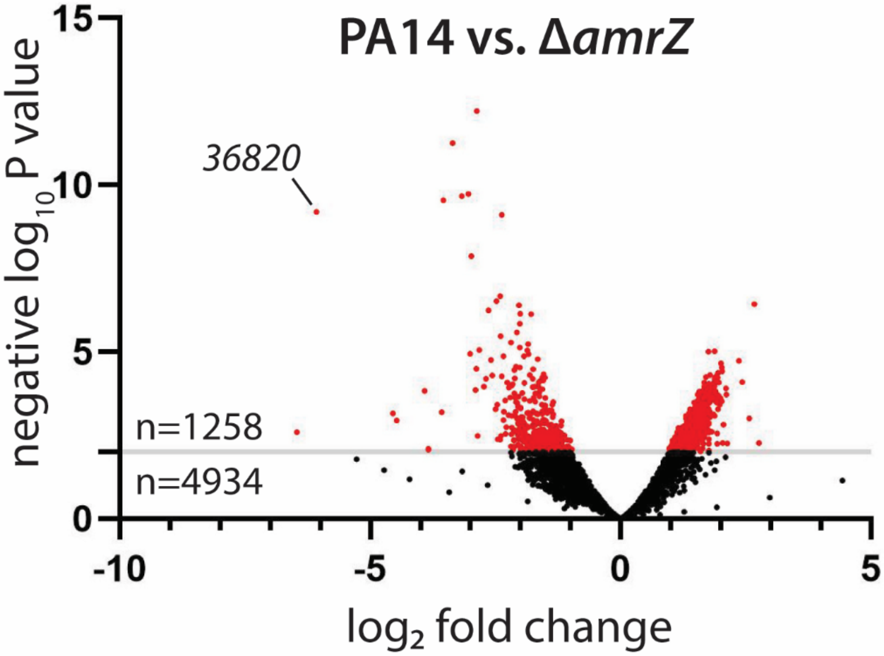

B

Locus tag	Log_2 fold change	Negative log_{10} p value
PA14_20480	-1.491	1.628
PA14_28600	-1.246	0.695
PA14_36820	-6.072	9.19
PA14_49300	1.342	1.679
PA14_49310	1.692	1.319
PA14_72360	1.322	1.029

Figure S2

Figure S2. Position of 16550-regulated genes in transcriptional comparison of wild-type and Δ*amrZ* strains. (A) Volcano plot of differentially regulated genes in a Δ*amrZ* strain versus wild-type PA14. A P-value of 10^{-2} was set as the significance threshold (grey horizontal line). The position of the *36820* gene in the plot is labeled, as it is the only significantly differentially regulated gene among the six identified 16550-regulated genes. (B) Table indicating the fold changes and P-values of the 6 16550-regulated genes described in the main text.

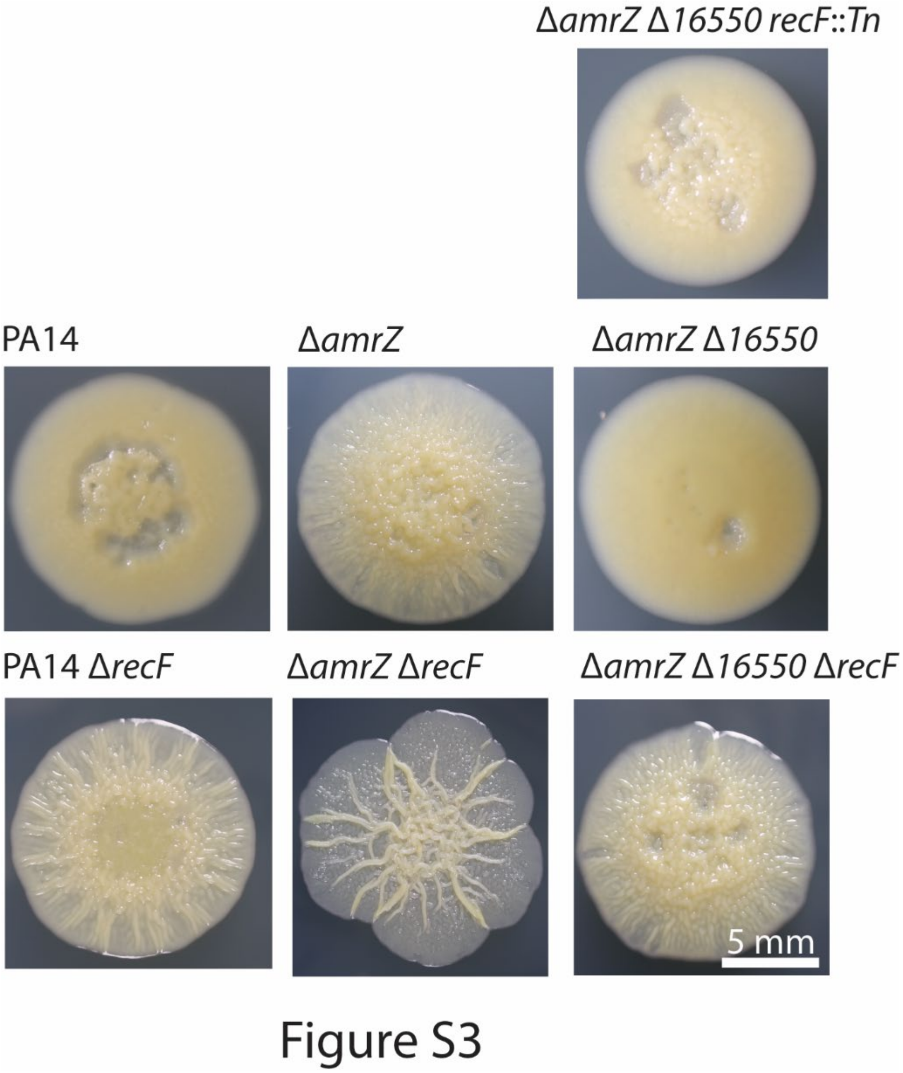

Figure S3. Effect of *recF* interruption or deletion on biofilm phenotypes.

Representative photographs of colony morphology after 6 d of growth at 25°C on M6301 agar of strains as indicated.

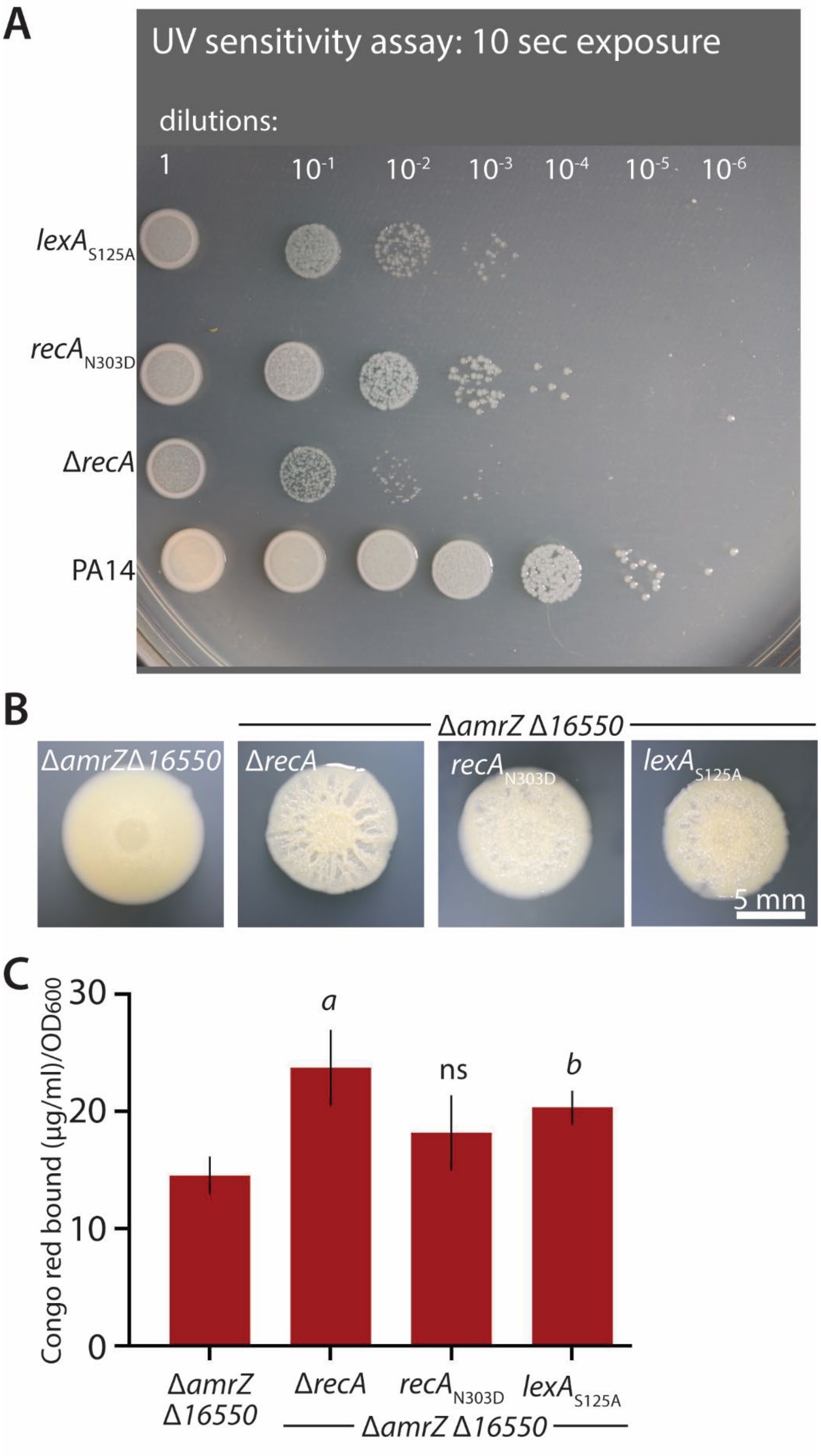
A
UV sensitivity assay: 10 sec exposure
dilutions:
1
10-1
10-2
10-3
10-4
10-5
10-6
lexAS125A
recAN303D
ΔrecA
PA14
B
ΔamrZ Δ16550
ΔamrZΔ16550
ΔrecA
recAN303D
lexAS125A
5 mm
C
Congo red bound (µg/ml)/OD600
30
20
10
0
a
ns
b
ΔamrZ Δ16550
ΔrecA
recAN303D
lexAS125A
ΔamrZ Δ16550

Figure S4

Figure S4. UV sensitivity of RecA and LexA substitution mutants and their biofilm phenotypes in a Δ*amrZ* Δ*16550* background. (A) Representative photograph of dilutions of the indicated strains treated for 10 s with ultraviolet light in a Stratalinker (see Methods). (B) Representative photographs of colony morphology of the indicated strains after 6 d of growth at 25°C on M6301 agar. (C) Congo red binding (normalized to OD_{600}) by the indicated mutant strains from panel B. Mean values of at least three replicates are shown; error bars indicate ± 1 standard deviation. P-values, Student's t-test, vs. Δ*amrZ* Δ*16550*: *a*, P = 0.0004; *b*, 0.0003; ns, not significant (P = 0.05).

Table S1. *Escherichia coli* strains used in this study.

MTC27	SM10 (*F- endA1 hsdR17 supE44 thi-1 λ- recA1 gyrA96 relA1*); *E. coli* mating strain for conjugation with *P. aeruginosa*	[1]
MTC33	SM10 pBT24 (mating strain for Tn mutagenesis), $Gent_R$	[2]
MTC570	SM10 pEXG2-Δ*amrZ*, $Gent^R$	[3]
MTC1346	SM10 pEXG2-Δ*69700*, $Gent^R$	[3]
MTC2179	SM10 pEXG2-Δ*recA*	This study
CSS539	SM10 pEXG2-Δ*recF*	This study
CSS803	SM10 pEXG2-Δ*36820*	This study
CSS710	SM10 pEXG2-Δ*49310*	This study
CSS932	SM10 pEXG2-Δ*49300*	This study
CSS943	SM10 pEXG2-Δ*20480*	This study
CSS945	SM10 pEXG2-Δ*28600*	This study
CSS1458	SM10 pJN105-*36820*	This study
CSS1469	SM10 pEXG2-Δ*72360*	This study
CSS1702	SM10 pEXG2-Δ*49300-10*	This study
MTC2251	SM10 pEXG2-$recA_{N303D}$	This study
MTC2642	SM10 pEXG2-$lexA_{S125A}$	This study
MTC2641	SM10 pCTX-1-*recA*	This study

Table S2. Plasmids used in this study.

Plasmid	Description	Source or reference
pCTX-1	mini-CTX-1, integrative tet^R plasmid for *P. aeruginosa*	[4]
pCTX-1-*recA*	CTX-1 vector containing *recA*	This study
pEXG2	Integrating suicide plasmid for *P. aeruginosa*, gent^R, with sucrose counterselection	[5]
pEXG2-Δ*recA*	EXG2 containing flanking sequences of *recA*	This study
pEXG2-Δ*recF*	EXG2 containing flanking sequences of *recF*	This study
pEXG2-Δ*36820*	EXG2 containing flanking sequences of *36820*	This study
pEXG2-Δ*49310*	EXG2 containing flanking sequences of *49310*	This study
pEXG2-Δ*49300*	EXG2 containing flanking sequences of *49300*	This study
pEXG2-Δ*49300-10*	EXG2 containing upstream flank of *49300* stitched to *49310* downstream flank	This study
pEXG2-Δ*20480*	EXG2 containing flanking sequences of *20480*	This study
pEXG2-Δ*28600*	EXG2 containing flanking sequences of *28600*	This study
pEXG2-Δ*72360*	EXG2 containing flanking sequences of *72360*	This study
pEXG2-$recA_{N303D}$	EXG2 containing sequence encoding a mutation at the RecA asparagine (303) to aspartic acid.	This study
pEXG2-$lexA_{S125A}$	EXG2 containing sequence encoding a mutation at the LexA active-site serine residue (125) to alanine.	This study
pJN105-*36820*	pJN105, plasmid containing arabinose inducible promoter for *P. aeruginosa*	This study

Table S3. Primers used in this study.

NB: The listed primer sequences may include 5' overlaps for isothermal assembly and/or stitch PCR (in boldface type). The 3' end is complementary to the target genomic sequence.

Primer number and name	Sequence (5'-3')
Rnd1-ARB1	GGCCACGCGTCGACTAGTACNNNNNNNNNNNAGAG
Rnd1-ARB2	GGCCACGCGTCGACTAGTACNNNNNNNNNNNACGCC
Rnd1-ARB3	GGCCACGCGTCGACTAGTACNNNNNNNNNNNGATAT
Rnd1-TnM20	TATAATGTGTGGAATTGTGAGCGG
Rnd2-ARB	GGCCACGCGTCGACTAGTAC
Rnd2-TnM20	ACAGGAAACAGGACTCTAGAGG
BT20TnMseq	CACCCAGCTTTCTTGTACAC

986/ EXG2_Eco_*recA*_up_F	**TGCGCACCCGTGGAAATTAATTAAGGTACCGAATTC** GATGGAGTCCGACCTGACCG
987/*recA*_up_R	**TCGCCATTGG** TGAAGTCCTCGCGAAGTCAG
988/*recA*_down_F	**GAGGACTTCA** CCAATGGCGACCGTGCTCGATAC
989/ EXG2_Hind_*recA*_down_R	**TTATACGAGCCGGAAGCATAAATGTAAAGCAAGCTT**GACGAACCGGAGTTCCGCAAC
1013/EXG2_Eco_*49310*_up_F	**TGCGCACCCGTGGAAATTAATTAAGGTACCGAATTC**CAGTCGTTCAGCTACCGCATC
1014/*49310*_up_R	**CAACAGGTTGCG** GTCTGAATCTCCACGTATTCAAATG
1015/ *49310*_down_F	**GGAGATTCAGAC** CGCAACCTGTTGAAAGAGCG
1016/EXG2_Hind_*49310*_down_R	**TGCGCACCCGTGGAAATTAATTAAGGTACCGAATTC**ATGAACCAGACCGTCGCCAT
1033/EXG2_Eco_*36820*_up_F	**TGCGCACCCGTGGAAATTAATTAAGGTACCGAATTC** GTCCACGTTGCAGTCCAGGC
1034/ *36820*_up_R	**CACTGCGCGG** CAGAACAGATTTCATGGACGC
1035/ 36820_down_F	**ATCTGTTCTG** CCGCGCAGTGAAGTGAATGG
1036/EXG2_Hind_*36820*_down_R	**TTATACGAGCCGGAAGCATAAATGTAAAGCAAGCTT**GATCGCATCCTGGATGAAGAAC
1206/EXG2_Eco_*20480*_up_F	**TGCGCACCCGTGGAAATTAATTAAGGTACC GAATTC** ATGAACCAGACCGTCGCCAT
1207/*20480*_up_R	**GCTTGCTTCG** GTTCACCTCCATTTCGTAGCTTTGC
1208/*20480*_down_F	**GGAGGTGAAC**CGAAGCAAGCGCCGGGAGAC
1209/EXG2_Hind_*20480*_down_R	**TTATACGAGCCGGAAGCATAAATGTAAAGC AAGCTT** CATCGAATCGCTGCGTCCCC
1210/EXG2_Eco_*28600*_up_F	**TGCGCACCCGTGGAAATTAATTAAGGTACC GAATTC** AAAGGCCGGGATGACCTTCA
1211/*28600*_up_R	**TCCGGACGAA** GATCGTTTATCCCCTCTTGCATGAAAAC

1212/*28600*_Down_F	**ATAAACGATC** TTCGTCCGGAATCCTGGCG
1213/EXG2_Hind_*28600*_down_R	**TTATACGAGCCGGAAGCATAAATGTAA AGC AAGCTT** CCGAGTGACCGCGTTTCAGA
1214/EXG2_Eco_*49300*_up_F	**TGCGCACCCGTGGAAATTAATTAAGGT ACCGAATTC** GGGATGAAATCGGCTATTG C
1215/ *49300*_up_R	**TTTCTCCGTC** ATCATCGACTCCATCAAT GC
1216/ *49300*_Down_F	**AGTCGATGAT** GACGGAGAAAGAGGACG GGG
1217/EXG2_Hind_*49300*_down_R	**TTATACGAGCCGGAAGCATAAATGTAA AGCAAGCTT** CATCCTGCTGCTGTTCTTCG
1222/EXG2_Eco_$recA_{N303D}$_up_F	**TGCGCACCCGTGGAAATTAATTAAGGT ACCGAATTC** CCAGAAACAGGGCGCCACC
1223/$recA_{N303D}$_up_R	**ACTTGGCGGC** GTCCGCCTTGCCCTGGC
1224/$recA_{N303D}$_down_F	**CAAGGCGGAC**GCCGCCAAGTACCTGGAA GAC
1225/EXG2_Hind_$recA_{N303D}$_down_R	**TTATACGAGCCGGAAGCATAAATGTAA AGCAAGCTT** CTGCCGGAAAACTGGGACT GAAG
1226/ EXG2_Eco_$lexA_{S125A}$_up_F	**TGCGCACCCGTGGAAATTAATTAAGGT ACCGAATTC** ATCGAGAATGCGTTCGACG GTTT
1227/$lexA_{S125A}$_up_R	**TGTCCTTC** ATGGCCATGCCGCGCAC
1228/$lexA_{S125A}$_down_F	**CGCGGCATG** GCCATGAAGGACATCGGC ATTC
1229/EXG2_Hind_$lexA_{S125A}$_down_R	**TTATACGAGCCGGAAGCATAAATGTAA AGC AAGCTT** TTCCAGCCAACTGACCACGGT
1029/EXG2_Eco_*72370*_up_F	**TGCGCACCCGTGGAAATTAATTAAGGT ACC GAATTC** CATTCGGCTTCTGGGTTGTCCC
1030/*72370*_up_R	**AAGCCCGTTC** CGCTTTCTCCTTGGCATCCGG
1031/*72370*_down_F	**GGAGAAAGCG** GAACGGGCTTGCGTGACACC

1032/EXG2_Hind_*72370*_down_R	**TTATACGAGCCGGAAGCATAAATGTAA AGC AAGCTT** TGCCGTAAGTGACTGCCCCATG
1594/*36820*_FP_pJN105-Eco	**GTTTCTCCATACCCGTTTTTTTGGGCTA GC GAATTC** ATGGCACAGCATCAAGGTGG
1595/*36820*_RP_pJN105-Xba	**GAATTGGAGCTCCACCGCGGTGGCGGC CGC TCTAGA** GTTATCAGTTCCCGCCGTGG
891/EXG2_Eco_*recF*_up_F	**TGCGCACCCGTGGAAATTAATTAAGGT ACC GAATTC** GGAAGTGAACGGCGGCAC
1608/49300-10_up_R	**TCTTTCTCCGTC** GTCTGAATCTCCACGTATTCAAATG
1609/PA14_49300-10_Down_F	**GAGATTCAGAC** GACGGAGAAAGAGGACGGGG
892/*recF*_up_R	**GCAGGTGATG** TCAGTGTGATTAGAGGCGCATC
893/*recF*_down_F	**ATCACACTGA** CATCACCTGCGTGGACCCG
894/EXG2_Hind_*recF*_down_R	**TTATACGAGCCGGAAGCATAAATGTAA AGC AAGCTT** GCCATCGGTCTCGCCCAC

Modes of strain construction

CSS310

This strain was constructed from the transposon mutagenesis experiment. The location of the transposon was identified by sequencing and described in the materials and methods.

CSS260

This strain was constructed from the transposon mutagenesis experiment. The location of the transposon was identified by sequencing and described in the materials and methods.

CSS788 and **CSS790**

CSS788 and CSS790 were mated with CSS693: 100 µL of an overnight LB culture of CSS788 or CSS790 was spot-dried on LB plate, and 500µL of an overnight LB culture of CSS693 was subsequently dried on top of the first spot. The plate was then incubated at 37°C overnight, and the mating matrix was scraped up with a sterile spatula or loop and resuspended in 1000 µL sterile LB media. 100 µL of the suspension was spread on LB agar containing 75 µg/ml gentamycin and 25 µg/ml irgasan to select for *P. aeruginosa* transformants with integrated pEXG2-*ΔrecA*(Δ*PA14_recA*) plasmid. 4-6 colonies were then inoculated into 3ml of plain LB and grown at 37°C for 6 hours to accumulate second crossovers. Aliquots (typically 10µL and 20µL) of the LB culture were then spread on LB plates containing 6% sucrose to select against the plasmid. A number of the sucrose-resistant colonies arising were then patched on LB and LB with 20 µg/mL gentamycin. At least 2 sucrose-resistant, gent-sensitive clones were then streaked

for single colonies, checked by PCR for presence of the desired deletion, and frozen at -80°C in 50% glycerol.

CSS1108 and CSS110

CSS788 and CSS790 were mated with CSS1088 individually as described above for CSS693, and a 10-μL aliquot of the LB-resuspended mating mix was spread on LB plates with 75 μg/mL tetracycline and 25 μg/mL irgasan to select for *P. aeruginosa* transformants. At least 2 colonies were then re-streaked for single colonies on LB-tet (25 μg/mL), grown in LB overnight at 37°C, and stored in 50% glycerol at -80°C.

CSS1472 and **CSS1473**

CSS1472 and CSS1473were mated with CSS1458 individually as described above for CSS693, and a 10-μL aliquot of the LB-resuspended mating mix was spread on LB plates with 75 μg/mL gentamycin and 25 μg/mL irgasan to select for *P. aeruginosa* transformants. At least 2 colonies were then re-streaked for single colonies on LB-gent (20μg/mL), grown in LB overnight at 37°C, and stored in 50% glycerol at -80°C.

CSS632, CSS633and CSS664

Constructed like **CSS693**, but were mated with **CSS539**.

CSS818, CSS824, and CSS825

Constructed like **CSS693**, but were mated with **CSS803**.

CSS740 and CSS741

Constructed like **CSS693**, but were mated with **CSS710**.

CSS260

CSS997 and CSS995

Constructed like **CSS693,** but were mated with **CSS943.**

CSS1202 and CSS985

Constructed like **CSS693,** but were mated with **CSS953.**

CSS987 and CSS1315

Constructed like **CSS693,** but was mated with **CSS956**.

CSS959 and CSS976

Constructed like **CSS693,** but were mated with **CSS932**.

CSS1604 and **CSS978**

Constructed like **CSS693,** but were mated with **CSS945**

CSS1517 and **CSS1518**

Constructed like **CSS693,** but were mated with **CSS1469**

CSS1472 and **CSS1473**

E. coli strains **CSS539, CSS693, 710, 803, 932, 943, 953, 956, 1469**
The appropriate pEXG2-derived knockout plasmids (listed in Table S2) were electroporated into SM10 (MTC27), and transformants were selected on LB plates containing 20 μg/mL gentamycin.
CSS1088
The appropriate mini-CTX-1 (listed in Table S2) were electroporated into SM10 (MTC27), and transformants were selected on LB plates containing 25 μg/mL tetracycline.
CSS1458
The appropriate pJN105 (listed in Table S2) were electroporated into SM10 (MTC27), and transformants were selected on LB plates containing 20 μg/mL gentamycin.

Modes of plasmid construction
All plasmids constructed in this study were assembled from purified PCR products (using the primers listed in Table S3) and restriction enzyme-cleaved plasmid backbones by using isothermal assembly [6]. The insert sequences of all plasmids were verified via Sanger sequencing before they were used to create new strains.
pCTX-1-*recA*
The *recA* gene was PCR-amplified from PA14 genomic DNA using primers 1276 and 1277 and assembled into EcoRI/ Hind III-cleaved pCTX-1
pEXG2-Δ*recA*
The upstream and downstream flanking sequences of the *recA* coding sequence were amplified from PA14 chromosomal DNA using primer pairs 986/987 and 988/989, respectively. A fragment containing the *recA* gene deletion was generated by stitch PCR using the initial fragments as self-priming templates with primers 986 and 989. The resulting deletion fragment was assembled into EcoRI/HindIII-cleaved pEXG2.
pJN105-*36820*
The *36820* gene was PCR-amplified from PA14 genomic DNA using primers 1594 and 1595 and assembled into EcoRI/ XbaI-cleaved pJN105

pEXG2-Δ*36820*
The upstream and downstream flanking sequences of the *36820* coding sequence were amplified from PA14 chromosomal DNA using primer pairs 1033/1034 and 1035/1036, respectively. A fragment containing the *36820* gene deletion was generated by stitch PCR using the initial fragments as self-priming templates with primers 1033 and 1036. The resulting deletion fragment was assembled into EcoRI/HindIII-cleaved pEXG2.
pEXG2-Δ*49310*
The upstream and downstream flanking sequences of the *PA14_49310* coding sequence were amplified from PA14 chromosomal DNA using primer pairs 1013/1014 and 1015/1016,

respectively. A fragment containing the *49310* gene deletion was generated by stitch PCR using the initial fragments as self-priming templates with primers 1013 and 1016. The resulting deletion fragment was assembled into EcoRI/HindIII-cleaved pEXG2.

pEXG2-Δ*20480*

The upstream and downstream flanking sequences of the *PA14_20480* coding sequence were amplified from PA14 chromosomal DNA using primer pairs 1206/1207 and 1208/1209, respectively. A fragment containing the *20480* gene deletion was generated by stitch PCR using the initial fragments as self-priming templates with primers 1206 and 1209. The resulting deletion fragment was assembled into EcoRI/HindIII-cleaved pEXG2.

pEXG2- Δ*49300*

The upstream and downstream flanking sequences of the *PA14_49300* coding sequence were amplified from PA14 chromosomal DNA using primer pairs 1214/1215 and 1216/1217, respectively. A fragment containing the *49300* gene deletion was generated by stitch PCR using the initial fragments as self-priming templates with primers 1214 and 1217. The resulting deletion fragment was assembled into EcoRI/HindIII-cleaved pEXG2.

pEXG2- Δ*49300-10*

The upstream and downstream flanking sequences of the *PA14_49300-10* coding sequences (comprising two neighboring genes) were amplified from PA14 chromosomal DNA using primer pairs 1013/1608 and 1609/1217, respectively. A fragment containing the *49300* gene deletion was generated by stitch PCR using the initial fragments as self-priming templates with primers 1013 and 1217. The resulting deletion fragment was assembled into EcoRI/HindIII-cleaved pEXG2.

pEXG2-$recA_{N303D}$

A version of *recA* encoding the N303D substitution in RecA and flanking sequences (400-800 bp) on either side of the substitution was generated by using primers 1222/1223 for the upstream flank and 1224/1225 for the downstream flank. The full fragment was generated by stitch PCR using the initial fragments as self-priming templates with primers 1222 and 1225. The resulting $recA_{N303D}$ fragment was assembled into EcoRI/HindIII-cleaved pEXG2.

pEXG2-$lexA_{S125A}$

A version of *lesxA* encoding the S125A substitution in LexA and flanking sequences (400-800 bp) on either side of the substitution was generated by using primers 1226/1227 for the upstream flank and 1228/1229 for the downstream flank. The full fragment was generated by stitch PCR using the initial fragments as self-priming templates with primers 1226 and 1229. The resulting $lexA_{S125A}$ fragment was assembled into EcoRI/HindIII-cleaved pEXG2.

pEXG2- Δ*28600*

The upstream and downstream flanking sequences of the *PA14_28600* coding sequence were amplified from PA14 chromosomal DNA using primer pairs 1210/1211and 1212/1213, respectively. A fragment containing the *28600* gene deletion was generated by stitch PCR using

the initial fragments as self-priming templates with primers 1210 and 1213. The resulting deletion fragment was assembled into EcoRI/HindIII-cleaved pEXG2.

pEXG2- Δ*72360*

The upstream and downstream flanking sequences of the *PA14_72360*coding sequence were amplified from PA14 chromosomal DNA using primer pairs 1029/1030and 1031/1032, respectively. A fragment containing the *72360* gene deletion was generated by stitch PCR using the initial fragments as self-priming templates with primers 1029 and 1032. The resulting deletion fragment was assembled into EcoRI/HindIII-cleaved pEXG2

References

1. Simon, R., U. Priefer, and A. Pühler, *A Broad Host Range Mobilization System for In Vivo Genetic Engineering: Transposon Mutagenesis in Gram Negative Bacteria.* Bio/Technology, 1983. **1**(9): p. 784-791.
2. Kulasekara, H.D., et al., *A novel two-component system controls the expression of Pseudomonas aeruginosa fimbrial cup genes.* Mol Microbiol, 2005. **55**(2): p. 368-80.
3. Cabeen, M.T., S.A. Leiman, and R. Losick, *Colony-morphology screening uncovers a role for the Pseudomonas aeruginosa nitrogen-related phosphotransferase system in biofilm formation.* Mol Microbiol, 2016. **99**(3): p. 557-70.
4. Hoang, T.T., et al., *Integration-proficient plasmids for Pseudomonas aeruginosa: site-specific integration and use for engineering of reporter and expression strains.* Plasmid, 2000. **43**(1): p. 59-72.
5. Rietsch, A., et al., *ExsE, a secreted regulator of type III secretion genes in Pseudomonas aeruginosa.* Proc Natl Acad Sci U S A, 2005. **102**(22): p. 8006-11.
6. Gibson, D.G., et al., *Enzymatic assembly of DNA molecules up to several hundred kilobases.* Nat Methods, 2009. **6**(5): p. 343-5.
